# Risk-based selection for carotid revascularisation using the IMPROVE score versus standard care in symptomatic carotid artery disease: a model-based cost-effectiveness analysis using pooled-data

**DOI:** 10.1136/bmjopen-2025-114391

**Published:** 2026-05-27

**Authors:** Kelly P H Nies, Bram Ramaekers, Juul Bierens, Dorothee P Auer, Andreas Schindler, Tobias Saam, Daniel Bos, Pim A de Jong, Paul J Nederkoorn, Gert J de Borst, Robert J van Oostenbrugge, Manuela A Joore, M Eline Kooi, Luc J M Smits

**Affiliations:** 1 Department of Radiology and Nuclear Medicine, Maastricht University Medical Centre+, Maastricht, LI, Netherlands; 2 CARIM Cardiovascular Research Institute Maastricht, Maastricht University, Maastricht, LI, Netherlands; 3 Department of Clinical Epidemiology and Medical Technology Assessment, Maastricht University, Maastricht, LI, Netherlands; 4 Sir Peter Mansfield Imaging Centre and Mental Health & Clinical Neuroscience Unit, School of Medicine, University of Nottingham, Nottingham, Nottinghamshire, UK; 5 NIHR Nottingham Biomedical Research Centre, University of Nottingham, Nottingham, England, UK; 6 Institute of Neuroradiology, University Hospital, LMU Munich, Munich, BY, Germany; 7 Die Radiologie, Radiologie Rosenheim, Rosenheim, BY, Germany; 8 Department of Radiology and Nuclear Medicine, Erasmus MC University Medical Center Rotterdam, Rotterdam, ZH, Netherlands; 9 Department of Epidemiology, Erasmus MC University Medical Center Rotterdam, Rotterdam, ZH, Netherlands; 10 Department of Radiology and Nuclear Medicine, University Medical Centre Utrecht, Utrecht, GE, Netherlands; 11 Department of Neurology, Amsterdam University Medical Centres, Duivendrecht, Noord-Holland, Netherlands; 12 Department of Vascular Surgery, University Medical Centre Utrecht, Utrecht, Netherlands; 13 Reinier de Graaf Gasthuis, Delft, Netherlands; 14 Department of Neurology, Maastricht University Medical Centre+, Maastricht, LI, Netherlands; 15 Department of Epidemiology, Maastricht University, Maastricht, Limburg, Netherlands

**Keywords:** HEALTH ECONOMICS, Stroke, Vascular surgery, Health Care Costs

## Abstract

**Abstract:**

**Background:**

A clinical prediction model (IMPROVE) for ipsilateral ischaemic stroke risk in symptomatic patients with carotid disease was recently developed with good performance. We aim to evaluate the model-based cost-effectiveness of IMPROVE-based triage versus triage in care-as-usual (CAU) for optimal medical treatment (OMT) alone or carotid endarterectomy plus OMT.

**Methods:**

A dataset of 678 patients with carotid disease and a recent ipsilateral ischaemic stroke, transient ischaemic attack or amaurosis fugax from four cohort studies informed a decision-analytic model. Stratification of patients for carotid endarterectomy was based on ≥50% carotid stenosis (CAU arm) or a range of 3-year ipsilateral ischaemic stroke risk thresholds (IMPROVE arm). The threshold resulting in the lowest number of ipsilateral strokes and perioperative strokes and deaths was selected as the optimal threshold. Patients with <50% stenosis (CAU) or an IMPROVE risk score below the threshold (IMPROVE) were modelled to receive OMT only. Parameter uncertainty was incorporated in probabilistic analyses using Monte Carlo simulations for a 3-year and lifetime horizon. Subgroup analyses for <50%, 50–69% and 70–99% carotid stenosis were performed.

**Results:**

IMPROVE-based triage reduced ipsilateral ischaemic strokes and perioperative strokes and deaths by 34.5% (CAU: 4.3%, IMPROVE: 2.8%) over 3 years. Revascularisations decreased by 20% with IMPROVE, while Quality-Adjusted Life Years slightly increased. Procedural stroke occurred in 1.8% of patients in CAU versus 1.4% of patients for IMPROVE. Societal costs decreased on average by €1441/patient for IMPROVE versus CAU for a 3-year time horizon (lifetime cost reduction: €6101/patient). Subgroup analyses identified IMPROVE as the superior strategy for 50–69% and 70–99% stenosis (3-year and lifetime horizon) and <50% stenosis (lifetime horizon).

**Conclusions:**

In this modelling analysis, triage of symptomatic patients with carotid disease with the IMPROVE model can lead to the prevention of one-third of ipsilateral ischaemic strokes and perioperative strokes and deaths, while also reducing societal costs. These findings should be validated in a clinical trial.

**STRENGTHS AND LIMITATIONS**

- A pooled dataset from four cohort studies enabled direct comparison of care-as-usual and IMPROVE strategies in a representative target population.
- Cost-effectiveness was assessed from a societal perspective over both 3-year and lifetime horizons, capturing long-term economic impact.
- Probabilistic sensitivity analyses (10 000 Monte Carlo simulations) and scenario analyses supported robustness across surgical risk and stenosis subgroups.
- Healthcare and indirect societal costs could not be disaggregated due to limitations in the available cost data.

## Introduction

 Patients with symptomatic carotid artery stenosis face a substantial risk of recurrent ipsilateral ischaemic stroke.^1^ Current guidelines recommend revascularisation only for patients with ≥50% carotid stenosis. However, patients with mild (20–49%) stenosis still have a substantial risk of 7.4% for an ipsilateral ischaemic stroke or transient ischaemic attack (TIA) within 3 years.^2 3^ The guidelines for selecting patients for revascularisation are based on findings from trials in the 1980s and 1990s.^4^ Since then, substantial improvements in optimal medical treatment (OMT) and adherence to OMT have occurred. Also, non-invasive imaging methods of carotid plaques have been developed and extensively validated since these trials were performed. It is evident that plaque vulnerability, besides the degree of stenosis, is a main factor driving ipsilateral ischaemic stroke recurrence. Plaque vulnerability markers, such as the presence of intraplaque haemorrhage (IPH) on MRI, indicate a sixfold to tenfold increased chance of ipsilateral ischaemic stroke.^5^

The IMPROVE clinical prediction model, developed to provide up-to-date ipsilateral ischaemic stroke risk estimates under OMT, includes five predictors: age, sex, the degree of carotid stenosis (<50%, 50–69%, 70–99%), the classification of the last event (ocular vs cerebral) and the presence of IPH as identified on MRI.^6^ Conditional stroke probability was applied to account for the stroke-free interval between the index event and risk estimation. The score showed good discrimination (C-statistic: 0.82 after internal validation) and a good calibration with a slope of 0.93.

The IMPROVE model could be used to select symptomatic patients at high risk for recurrent stroke for carotid revascularisation. Since these procedures carry a 2.7–4.8% risk of procedural stroke or death, the IMPROVE model can also be used to select patients with a lower risk for recurrent stroke for OMT only.^7^ The optimal threshold to stratify patients for revascularisation needs to be examined to correctly balance the selection of individuals for revascularisation with high risk of stroke recurrence while also avoiding this potentially risky procedure in patients with lower risk profile.

Empirical impact studies randomising patients to care-as-usual (CAU) or prediction-based care are costly and time-intensive. Decision-analytic models can provide preliminary insights into differences in health outcomes and costs between a care innovation (such as IMPROVE) and CAU and can inform the optimal risk threshold as well as the need and necessary design features of a subsequent empirical study.

We used a decision-analytic model to investigate the effect of selecting symptomatic patients with carotid artery disease for carotid revascularisation plus OMT versus OMT alone using different thresholds of the IMPROVE risk score on the number of strokes and cost-effectiveness using CAU as reference.

## Methods

### Study design

This study followed the guiding principles of the^8^Consolidated Health Economic Evaluation Reporting Standards (CHEERS) 2022, CHEERS 2022 (online supplemental table 1).^8^ Costs are estimated from a societal perspective in line with recommendations from the Dutch Manual for Cost Analysis in Health Care Research.^9^ The primary outcome is procedural stroke and death and ipsilateral ischaemic stroke during 3 years of follow-up. Additionally, Quality-Adjusted Life Years (QALYs) and costs are compared for the two selection strategies and the cost-effectiveness for a lifetime time horizon is estimated. We adopted a willingness-to-pay threshold of €50 000/QALY gain, set by the Dutch National Health Care Institute.^10^

**Figure 1:**
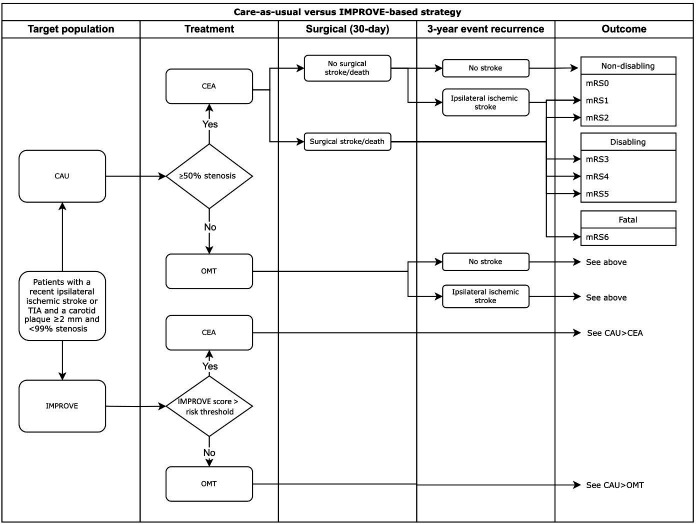
Decision-tree of care-as-usual (CAU) and an IMPROVE-based strategy of patient stratification for carotid endarterectomy (CEA). In CAU, all patients with ≥50% stenosis would undergo CEA, while patients in IMPROVE-based stratification would undergo CEA when their 3-year ipsilateral ischaemic stroke risk exceeded the threshold. For simplicity, only the CAU decision-tree is fully illustrated, since the branches are identical after risk stratification for CEA or optimal medical treatment (OMT) only. TIA, transient ischaemic attack.

This decision-analytic model employed a decision-tree cohort simulation (figure 1), informed by recent data from four cohorts of patients on OMT^11^,^14^ and contemporary registry data on perioperative strokes and deaths and ipsilateral stroke rate after revascularisation.^15^,^17^ For cohorts included in a previous analysis,^5^ individual consent was waived by the local ethics committee (Ethikkommission der Ludwig-Maximilian-Universität München). An additional cohort not included in a previous analysis^14^ received ethics approval (Medisch Ethische Commissie azM/UM, MEC 09–2-082), and all patients provided written informed consent.

#### Selection strategies

In CAU, all patients with ≥50% carotid stenosis were modelled to undergo carotid endarterectomy (CEA) plus OMT according to guidelines from the European Society for Vascular Surgery.^2^ Patients with <50% stenosis were modelled to undergo OMT only.

In the IMPROVE-based strategy, selection for CEA was based on the 3-year risk of ipsilateral ischaemic stroke determined using the IMPROVE risk score (box 1).^6^ Any missing predictor values were imputed using multiple imputation by chained equations by means of the ‘quickpred’ and ‘mice’ functions of the ‘mice’ package (V.3.16.0) in RStudio (V.4.2.0).

Consequently, in the IMPROVE-based strategy, patients exceeding the IMPROVE risk threshold were modelled to undergo CEA plus OMT. Patients with an ipsilateral ischaemic stroke risk below this threshold were modelled to undergo OMT only. The expected clinical impact of the IMPROVE model was evaluated for thresholds between 5% and 20% 3-year risk of ipsilateral ischaemic stroke. The IMPROVE-based stratification can diverge from stenosis-based stratification, potentially modelling some patients with <50% stenosis for CEA and modelling some patients with ≥50% stenosis for OMT only.

**Box 1 Formula for 3-year risk of ipsilateral ischaemic stroke in symptomatic patients with carotid artery disease according to the IMPROVE risk score, assuming a time since index event of no more than 9 days and therefore without conditional survival.^6^ Note that conditional stroke probability needs to be applied for time since index event for more than 9 days.**

Consequently, In the IMPROVE-based strategy, patients exceeding the IMPROVE risk threshold were modeled to undergo CEA plus OMT. Patients with an ipsilateral ischemic stroke risk below this threshold were modeled to undergo OMT only. Expected clinical impact of the IMPROVE model was evaluated for thresholds between 5 and 20% 3-year risk of ipsilateral ischemic stroke. The IMPROVE-based stratification can diverge from stenosis-based stratification, potentially modeling some patients with <50% stenosis for CEA and modeling some patients with ≥50% stenosis for OMT-only.

#### Study population

Both the CAU and IMPROVE-based strategies were applied to recent clinical data to generate insight into the number of patients stratified for CEA in combination with OMT versus OMT-only and corresponding perioperative stroke and perioperative death and ipsilateral ischaemic stroke-free survival probabilities. Data were pooled from four observational studies of recently symptomatic patients (ischaemic stroke, TIA or amaurosis fugax <6 months ago; n=678) with carotid artery disease who were on OMT and had a carotid MRI for IPH identification (table 1).^11^,^14^ Across all cohorts, IPH was imaged using a magnetisation-prepared rapid gradient-echo or similar sequence, in line with international recommendations for IPH imaging.^18^

**Table 1:** Clinical characteristics of the study population

Characteristic	Overall n=678	Hosseini, n=179	Hosseini, n=149	Kwee, n=126	van Dam-Nolen, n=224
Baseline characteristics					
Age	71±10	72±10	75±10	69±10	69±9
Male sex	450 (66%)	127 (71%)	88 (59%)	79 (63%)	156 (70%)
Index event					
TIA	282 (42%)	78 (44%)	58 (39%)	47 (37%)	99 (44%)
Stroke	299 (44%)	63 (35%)	73 (49%)	63 (50%)	100 (45%)
Amaurosis fugax	97 (14%)	38 (21%)	18 (12%)	16 (13%)	25 (11%)
Time since last event (in days)	37 (21–62)	42 (20–82)	24 (13–46)	30 (20–41)	48 (33–67)
Degree of stenosis					
0–49%	322 (50%)	0 (0%)	62 (42%)	82 (65%)	178 (95%)
50–69%	192 (30%)	68 (38%)	71 (48%)	44 (35%)	9 (4.8%)
70–99%	127 (20%)	111 (62%)	16 (11%)	0 (0%)	0 (0%)
IPH presence on MRI	292 (43%)	114 (64%)	54 (36%)	37 (29%)	87 (39%)
Current smoker	221 (33%)	106 (59%)	36 (24%)	29 (23%)	50 (22%)
Hypertension	455 (70%)	142 (79%)	121 (81%)	27 (21%)	165 (83%)
Diabetes mellitus	134 (20%)	20 (11%)	33 (22%)	31 (25%)	50 (25%)
History of ischaemic CVD	121 (31%)	Unknown	Unknown	31 (25%)	47 (21%)
Statin use before index event	441 (65%)	140 (78%)	76 (51%)	Unknown	109 (49%)
Follow-up information					
Follow-up (in months)	17.0 (10.6–59.2)	2.2 (0.8–15.2)	20.2 (11.3–32.0)	12*	61.7 (38.5–69.4)
Ipsilateral ischaemic cerebrovascular events	130 (19%)	62 (35%)	20 (13%)	13 (10%)	35 (16%)
Stroke	58 (8.6%)	26 (15%)	14 (9.4%)	3 (2.4%)	15 (6.7%)
TIA	53 (7.8%)	25 (14%)	3 (2.0%)	9 (7.1%)	16 (7.1%)
Amaurosis fugax	20 (2.9%)	11 (6.1%)	3 (2.0%)	1 (0.8%)	5 (2.2%)

Values are presented as n (%), mean±SD or median (IQR). Missing values include the degree of stenosis (n=37), hypertension (n=25), diabetes mellitus (n=20), time since last event (n=5) and statin use before index event (n=1).

* All patients had a 1-year follow-up.

CVD, cardiovascular disease; IPH, intraplaque haemorrhage; TIA, transient ischaemic attack.

OMT included lifestyle advice and medication including statin, antiplatelet and antihypertensive treatment. Ischaemic symptoms were related to the carotid artery territory and patients had an ipsilateral carotid plaque of ≥2 mm and a maximum of 99% carotid stenosis. The dataset was similar to the development dataset of the IMPROVE model. However, one study^19^ had an inclusion bias towards IPH-positive patients and was excluded in order to obtain overall ipsilateral ischaemic stroke risks that are representative of the target population. Patients with ≥70% stenosis, who would generally undergo carotid revascularisation considering current guidelines, were included in the dataset due to various reasons including patient preference for OMT only, existing comorbidities and delayed revascularisation procedures. Patients undergoing carotid revascularisation were censored at the time of the intervention.

The pooled dataset consisted of 678 recently symptomatic patients with carotid artery disease (table 1). Carotid stenosis of <50% was present in half of the study population and 30% and 20% of patients had 50–69% stenosis or 70–99% stenosis, respectively. The classification of the index event was primarily ischaemic stroke (44%) and TIA (42%) ipsilateral to the carotid plaque. In total, 130 ipsilateral cerebrovascular and ocular events, that is, 58 strokes, 53 TIAs and 20 amaurosis fugax, occurred during a median 17.0 (IQR: 10.6–59.2) months of follow-up. Patient characteristics were comparable to those in the European Carotid Surgery Trial (ECST), except for a higher median age, likely due to demographic shifts since the 1980 s–1990s.^20^ The cohort of 678 patients represented all available data from cohorts with both carotid MRI and clinical outcome data under OMT only. No formal sample size calculation was performed, as this was a model-based study using all eligible existing data rather than a prospective study powered to detect a difference between strategies.

### Input parameters regarding stroke risks, quality of life and costs

The input parameters for the decision-analytic study are provided in table 2. An overview of the sensitivities and specificities used for calculating the number of strokes is provided in online supplemental table 2.

**Table 2:** Input parameters for the decision-analytic study

Stroke risk (%) base value			Distribution	Source
Surgical	CEA surgical stroke or death (%)	3.6	Beta (235, 6224)	^15^
	Non-disabling surgical stroke (% of surgical events)	48.3	Dirichlet (43, 46)	^17^
	Disabling surgical stroke (% of surgical events)	38.2	Dirichlet (34, 55)	^17^
	Fatal surgical outcome (% of surgical events)	13.5	Dirichlet (12, 77)	^17^
	Remaining 3-year stroke risk after CEA	2.2	beta (166, 7423)	^16^
	Non-disabling stroke (% of post-surgical events)	50.0	Dirichlet (115, 115)	^17^
	Disabling stroke (% of post-surgical events)	34.8	Dirichlet (80, 150)	^17^
	Fatal stroke (% of post-surgical events)	15.2	Dirichlet (35, 195)	^17^
OMT	3-year stroke risk on OMT	Dependent on threshold*	Beta	Pooled dataset
	Non-disabling stroke	53.3	Dirichlet (81, 71)	^17^
	Disabling stroke	30.3	Dirichlet (46, 106)	^17^
	Fatal stroke	16.4	Dirichlet (25, 127)	^17^
Underlying assumptions for societal costs and utility				
mRS score categorisation	Amaurosis fugax/TIA	mRS 0		
	Non-disabling stroke	mRS 1–2	Dirichlet	^21^, online supplemental table 3
	Disabling stroke	mRS 3–5	Dirichlet	^21^, online supplemental table 3
	Fatal stroke	mRS 6		
Utility	Amaurosis fugax/TIA	0.960	Beta	^22^
	Non-disabling stroke (first event)	0.864	Beta	^22^
	Non-disabling stroke (recurrent event)	0.812	Beta	^22^
	Disabling stroke	0.356	Beta	^22^
	Fatal stroke	0		
Base utility	Weighted average of amaurosis fugax/TIA/non-disabling stroke (first event)	0.918	Beta	^22^
EVT	% of patients who received EVT	5.8%	Beta	^24^
Dutch costs (in 2022, valuta: €)				
Imaging	MRI	254	Gamma	^23^
Treatment	CEA	8733	Gamma	^23^
Societal	Non-disabling stroke (first 3 months)	14 511	Gamma	^22^
	Non-disabling stroke (cost/subsequent month)	999	Gamma	^22^
	Disabling stroke (first 3 months)	45 265	Gamma	^22^
	Disabling stroke (cost/subsequent month)	5865	Gamma	^22^
	Fatal stroke (one-time costs)	13 111	Gamma	^23^

* An overview of the sensitivities and specificities used for calculating the number of strokes is provided in online supplemental table 2.

CEA, carotid endarterectomy; CTA, computed tomography angiography; mRS, modified Rankin Score; OMT, optimal medical treatment; TIA, transient ischemic attack.

#### Ischaemic stroke risk estimation

Ipsilateral ischaemic stroke risks for intermediate-risk patients remaining on OMT were extracted from the pooled dataset. Kaplan-Meier estimations of 3-year ipsilateral ischaemic stroke-free survival in the intermediate-risk group informed the model about the number of expected strokes on OMT for CAU and IMPROVE. The sensitivity and specificity of the two strategies were calculated based on the numbers of patients correctly assigned to the high and intermediate risk groups, respectively, according to their ipsilateral stroke recurrence status.

A 3.6% 30-day procedural risk of any stroke and death as reported by the Dutch Audit of Carotid Interventions was used in the model for patients who were modelled to undergo CEA.^15^ Long-term post-revascularisation stroke risk on OMT was derived from registries.^16^

Strokes were categorised as non-disabling (Modified Rankin Scale (mRS) 1–2), disabling (mRS 3–5) or fatal, consistent with large clinical trials.^17^ Since the pooled dataset lacked information on stroke severity, proportions were modelled using ECST data.^17^ Proportions of stroke severity were modelled separately for OMT only and post-CEA using Dirichlet distributions. The proportion of mRS 1/mRS 2 for non-disabling and mRS 3–5 for disabling strokes was not reported in ECST and therefore based on an observational study reporting on mRS distribution at the time of hospital discharge.^21^

#### Quality of life inputs

A recent TIA or amaurosis fugax as index event had a baseline utility of 0.960, equivalent to mRS 0.^22^ Non-disabling stroke as the index event had a baseline utility of 0.864 /year, calculated as the weighted average of mRS 1 and 2 utilities observed at discharge.^21 22^ Recurrence of a non-disabling stroke reduced utility by 0.0524 /year,^23^ resulting in 0.812 /year. Disabling stroke had a utility of 0.356, the weighted average of mRS 3–5 utilities.^22^ Calculations of the weighted average utility input parameters for non-disabling and disabling stroke are included in online supplemental table 3.

Patients were assumed to remain in their baseline mRS category unless a procedural event or ischaemic stroke caused a change. CEA could lead to procedural stroke or death, with corresponding changes in mRS applied immediately. No additional penalty on utility was applied for the procedure itself. For patients on OMT who experienced ischaemic stroke recurrence, mRS changes occurred at the modelled recurrence time, determined by a gamma distribution with a mean of 24.1±2.7 months from the index event, based on the pooled observational dataset. The new mRS and associated utility were assumed to remain constant for the remainder of the time horizon.

#### Societal cost inputs

Costs of examinations included the costs for patients in the IMPROVE intervention who would need an additional MRI to determine the IMPROVE-based estimation of the risk of stroke. Only patients for whom the outcome of the MRI examination could potentially result in recategorisation from the revascularisation to the OMT-only group would undergo an MRI at the cost of €252 (table 2). The probability of a patient needing an MRI for IMPROVE categorisation depended on the IMPROVE threshold (online supplemental table 4). The cost of duplex ultrasound and CTA performed at the time of the index event was not considered since no differences in usage are expected for CAU and IMPROVE strategies. Cost of treatment included CEA for those selected at high risk and OMT costs for those with intermediate risk (table 2). CEA costs were incurred at the start of the time horizon.

The costs associated with stroke recurrence are determined by the severity of the stroke. Since all patients were symptomatic, a baseline cost level was applied, which was the weighted average of the index event considering the distribution of minor stroke, TIA and amaurosis fugax at baseline (table 2). During the first 3 months after the index event and any recurring event, the monthly costs peak due to the intensity of required medical care.^22^ Patients that did not experience event recurrence remained at the baseline cost level for the duration of the time horizon. The costs associated with a recurrent non-disabling stroke were determined based on the weighted average societal costs of mRS 1 and mRS 2, resulting in €14 511 total in the first 3 months and €999 during each following month (table 2, online supplemental table 3).^22^ Societal costs of a disabling stroke were determined using the distribution between mRS 3 and 5 and their corresponding reported costs, resulting in €45 265 for the first 3 months and €5865 for each subsequent month.^22^ Fatal strokes had a one-time cost of €13 111.^23^ Societal costs were split based on whether or not patients underwent endovascular treatment (EVT). We assumed that 5.8% of patients received EVT in order to determine the average societal cost per mRS score.^24^

Since the CAU and IMPROVE strategies lead to different probabilities of patients having fatal strokes, the average costs of the index event differed between CAU and IMPROVE. Since the costs of the index event are unpreventable, we subtracted the average cost of the index event from the total societal costs per strategy. Societal costs included healthcare, patient and family costs and other costs including productivity loss.^22 23^ All unit prices were converted to the year 2022 using the Dutch Consumer Price Index (CPI) (with a factor CPI 2022/CPI 2012=1.265).

### Analyses

#### Base case analysis

The primary analysis used a time horizon of 3 years. We calculated the total expected number of strokes and QALYs and the total expected costs without the costs of the index event for CAU and different thresholds of the IMRPOVE score. The average cost of the index event was excluded from the analysis to estimate the preventable costs of event recurrence only.

Probabilistic analyses incorporating uncertainty of parameter values were visualised on a cost-effectiveness plane. Monte Carlo simulations (n=10 000) were run and uncertainty of parameters was modelled using beta and Dirichlet distributions for binary and categorical parameters, respectively. Cost uncertainty was modelled using gamma distributions. Additional details on the distributions applied to each input variable are summarised in table 2. Tornado diagrams were used to illustrate the top 10 most influential parameters related to the numbers of strokes, QALYs and societal costs. All model analyses were performed in R (V.4.3.0) using the ‘dampack’ (V.1.0.1) and ‘BCEA’ (V.2.4.6) packages.

#### Scenario-analyses

Scenario-analyses were performed taking into account varying strategies of CAU stratification and surgical risks. Subgroup analyses per degree of stenosis were performed to ensure IMPROVE threshold selection was suitable for all degrees of stenosis. IMPROVE versus CAU was analysed for degree of stenosis subgroups of <50%, 50–69% and 70–99% stenosis. A difference in the timing of a new ipsilateral ischaemic stroke was expected for the varying subgroups, therefore the average time to stroke recurrence was reassessed for each group. Average time to recurrence was 34.2 (SD: 0.9) months for <50% stenosis, 24.2 (SD: 1.4) months for 50–69% stenosis and for 70–99% stenosis an average of 10.7 (SD: 1.2) months.

Another influential factor is the surgical risk of stroke or death. Two alternative scenarios were modelled: one in which this risk was set at 6%, representing the maximum accepted threshold for procedural risk, and one in which it was set at 2%, reflecting an optimistic scenario characterised by low perioperative risks.^2 25^ Since the benefit of carotid revascularisation is considered fourfold higher in patients with 70–99% stenosis versus patients with 50–69% stenosis, a scenario where only patients with 70–99% stenosis would undergo revascularisation was considered.

#### Long-term cost-effectiveness analyses

Since a major ischaemic stroke has life-long consequences, the 3-year time horizon will inherently underestimate QALY gain and the reduction in societal costs associated with the IMPROVE-directed treatment strategy in comparison to CAU. Ipsilateral ischaemic stroke-free survival on OMT was similar in patients with intermediate risk according to CAU and IMPROVE in the period from 3 years to 6 years after the index event (online supplemental figure 2). Therefore, the treatment selected based on the initial risk assessment no longer exerts a noticeable impact on ipsilateral ischaemic stroke recurrence and we assume that both CAU and IMPROVE strategies will be related to a similar probability of stroke recurrence from 3 years after the index event onwards. Since the average decline in quality of life after the 3-year time horizon is expected to be comparable for both strategies, we assumed that changes in mRS after 3 years will not contribute to the cost-effectiveness comparison of CAU and IMPROVE. To determine the QALY and costs for the life-long time horizon, mRS scores were assumed to remain stable from the end of the 3-year time horizon until the end of the life-long time horizon.

Life-long QALY and societal costs of CAU and IMPROVE were explored using a previously established stroke model which estimated societal costs associated with each mRS while taking into account the annual probability of mortality of the general Dutch population and a discount rate of 4% for costs and 1.5% for QALYs.^26^ Societal costs and utility per mRS score were adjusted to recently reported figures from 2022.^27^ The average life-long QALY and costs of each mRS score were modelled from a starting age of 74 years, since at baseline patients had a median age of 71 years and the QALYs and costs of the first 3-year post-index event were covered in our base model with a 3-year time horizon. Cycle length of the previously established stroke model was 1 year and mortality rates of the corresponding life-year were applied to the model.^26^ Life-long QALY ranged from 0.795 for mRS 5 to 7.771 for mRS 0, while societal costs were between €55 317 for mRS 0 and €1 041 095 for mRS 5 (online supplemental figure 2). The distribution of patients’ mRS at the end of the 3-year time horizon was established in our base model. Life-long QALY and societal costs were estimated by multiplying the average QALY and societal costs per mRS score to the number of patients per mRS score at the end of the 3-year time horizon. Results are presented as the average QALY and societal costs/person.

## Results

Stratification of patients at high risk using CAU criteria (≥50% stenosis) resulted in a sensitivity of 78.4% (95% CI 68.0 to 86.5) and specificity of 55.2% (95% CI 51.1 to 59.3) for recurrent ipsilateral stroke. The sensitivity of IMPROVE was higher compared with CAU for the 3-year risk thresholds of 5–11% (online supplemental figure 1). Both sensitivity and specificity were higher compared with CAU for IMPROVE risk thresholds of 9–11%.

### Base-case results

Probabilistic analyses of CAU versus IMPROVE resulted in a 24.2% decrease of the primary endpoint, that is, the number of ipsilateral ischaemic strokes and perioperative strokes and deaths for the IMPROVE threshold of 5% 3-year risk in comparison to CAU (online supplemental table 6). The IMPROVE strategy was analysed per 1% increase in threshold, reaching the maximum decrease in the primary endpoints of 34.5% with the 10% IMPROVE threshold. Use of a 20% IMPROVE threshold resulted in a 15.9% decrease in the primary endpoint. The IMPROVE strategy led to a marginal increase in the average QALY/person, which ranged between 0.007 for the 5% threshold and 0.016 for the 20% threshold of IMPROVE versus CAU.

Since the 10% 3-year risk threshold of IMPROVE provided the largest decrease in the number of ipsilateral ischaemic strokes, we have selected this threshold as our base-case. CAU-based selection for CEA resulted in 49% of patients to undergo surgery (332/678), whereas IMPROVE would select only 40% of patients (268/678) patients to undergo surgery, a 19% decrease. Out of the 678 patients in the simulation, on average 29.0 (95% CI 28.9 to 29.1) patients in the CAU-strategy and 19.1 (95% CI 19.0 to 19.2) patients in the IMPROVE-strategy had a new ipsilateral ischaemic stroke during the 3-year time horizon. Procedural stroke occurred in 12.1 (95% CI 12.1 to 12.1) (1.8%) patients for the CAU regimen and 9.7 (95% CI 9.7 to 9.8) (1.4%) patients for the IMPROVE regimen. New non-disabling (perioperative) strokes occurred in 14.8 (95% CI 14.8 to 14.9) (2.2%) patients for CAU versus 9.6 (95% CI 9.6 to 9.7) (1.4%) patients for IMPROVE. CAU was also accompanied by a higher frequency of major (9.8 (95% CI 9.8 to 9.8) versus 6.6 (95% CI 6.6 to 6.6) and fatal (4.4 (95% CI 4.4 to 4.4) versus 2.8 (95% CI 2.83 to 2.85)) strokes in comparison to IMPROVE. At the end of the 3-year time horizon, the model resulted in the following distribution of mRS for CAU versus IMPROVE, respectively: 362.7 versus 368.3 patients in mRS0, 216.8 versus 216.3 in mRS1, 84.2 versus 84.0 in mRS2, 2.2 versus 1.5 in mRS3, 5.2 versus 3.5 in mRS4, 2.4 versus 1.6 in mRS5 and 4.4 versus 2.8 in mRS6. The average QALY per patient at 3 years was 2.691 (95% CI 2.691 to 2.691) for IMPROVE, while CAU was associated with a QALY of 2.677 (95% CI 2.677 to 2.677). Average difference between QALYs of the two interventions was 0.014 (95% CI 0.014 to 0.014) in favour of IMPROVE.

Societal costs associated with CAU and IMPROVE based on the 10% 3-year risk threshold averaged to €6509 (95% CI €6474 to €6543) and €5123 (95% CI €5095 to 5151) per patient during the time horizon of 3 years, respectively. This resulted in a saving of €1386 (95% CI €1376 to €1396) per patient by IMPROVE. With a decrease in societal costs and increase in QALY, IMPROVE is considered the dominant strategy. Deterministic decision-analytic models confirmed the findings of the probabilistic models (online supplemental table 6).

### Sensitivity and scenario analyses

**Figure 2:**
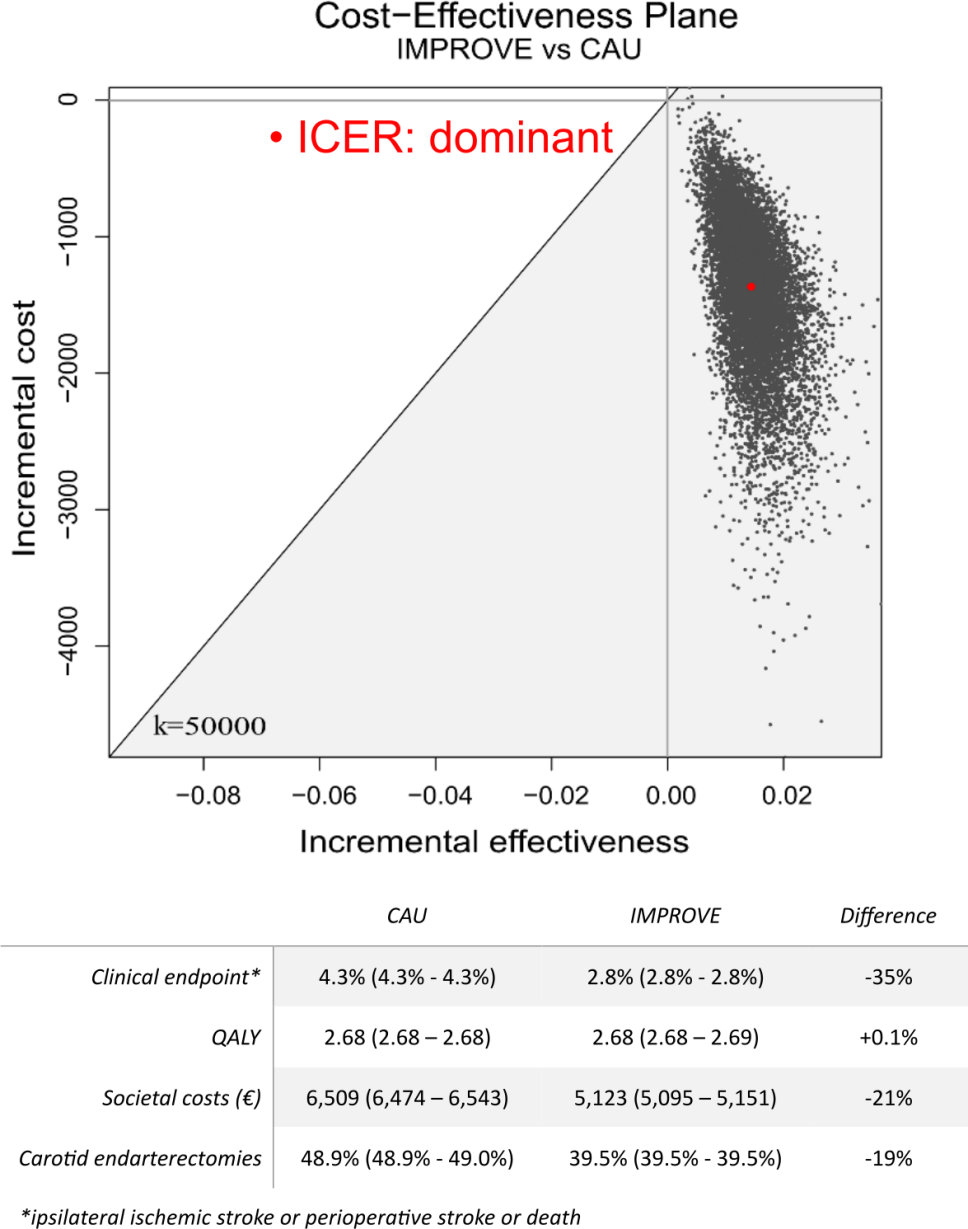
Cost-effectiveness plane of IMPROVE versus CAU with a 3-year time horizon. All 10 000 simulations indicated that IMPROVE with a 3-year ipsilateral ischaemic stroke risk threshold of 10% was the optimal strategy compared with CAU. Numbers of ipsilateral ischaemic strokes, QALY/patient, societal costs/patient and number of carotid endarterectomies are reported with 95% CIs. Index event costs were excluded. CAU, care-as-usual; ICER, incremental cost-effectiveness ratio; Quality-Adjusted Life Year, QALY.

Considering the willingness-to-pay threshold of €50 000 per gained QALY, 100% of simulations of IMPROVE with a threshold of 10% 3-year ipsilateral ischaemic stroke were cost-effective compared with CAU (figure 2). All probabilistic simulations identified an increase in the average QALY for IMPROVE and 99.95% of simulations resulted in a decrease in societal costs for a 3-year time horizon.

**Figure 3:**
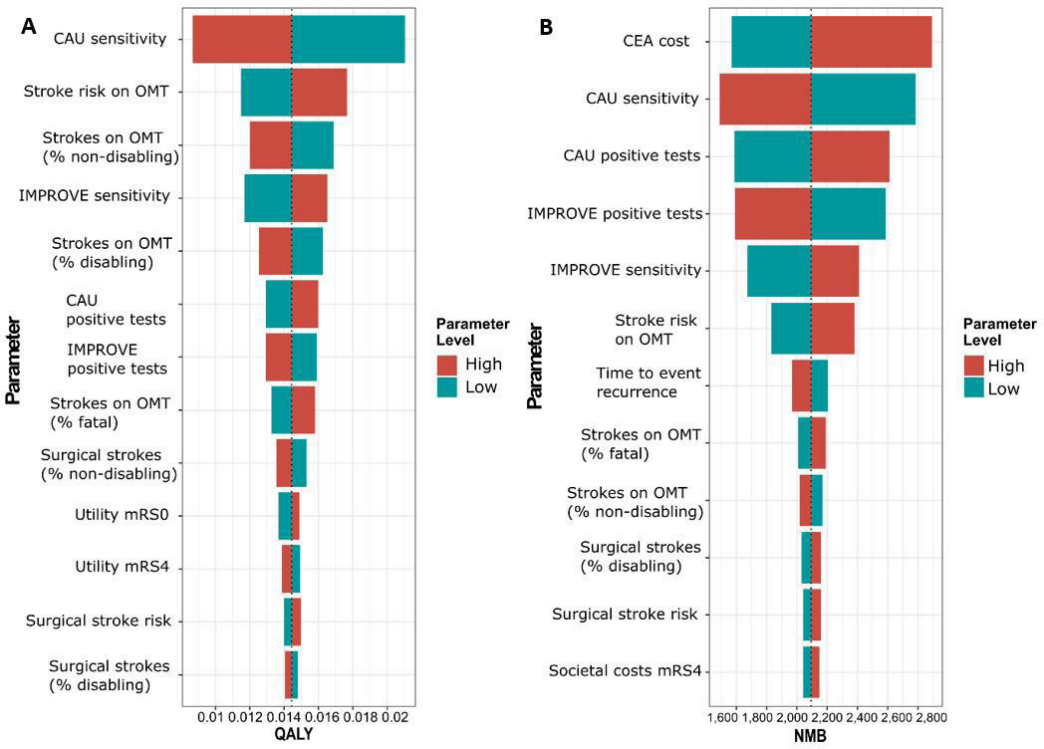
Probabilistic sensitivity analysis of QALY and societal costs of IMPROVE versus care as usual. (**A**) Tornado graph of input parameters leading to a >5% change in societal costs of IMPROVE versus care as usual. (**B**) Tornado graph of input parameters leading to a >5% change in net monetary benefit (NMB)/patient of IMPROVE versus care as usual. In all sensitivity analyses, IMPROVE was the dominant strategy considering that both QALY and NMB were higher for an IMPROVE-based strategy compared with care as usual. CAU, care as usual; CEA, carotid endarterectomy; mRS, modified Rankin Score; OMT, optimal medical treatment; Quality-Adjusted Life Year, QALY.

Univariate deterministic sensitivity analyses demonstrated that the most influential parameter on the QALY outcome was the sensitivity of CAU to detect patients who will experience a new ipsilateral ischaemic stroke (figure 3). A change in CAU sensitivity resulted in a QALY gain between 0.009 and 0.021 in favour of IMPROVE. Other influential parameters on QALY outcome consisted of stroke risk on OMT and procedural stroke risks including the distribution between non-disabling, disabling and fatal strokes. The most influential parameter on the net monetary benefit (NMB) was the cost of CEA with an increase in NMB for IMPROVE ranging from €1598 to €2871 (figure 3). No singular parameter caused a change in optimal strategy from IMPROVE to CAU.

Carotid revascularisation by means of CEA is recommended up to an expected 6% 30-day stroke/death risk in the guidelines of the European Society for Vascular Surgery.^2^ An alternative scenario analysis with a 6% surgical stroke/death risk resulted in 2.664 QALY for CAU and 2.681 QALY for IMPROVE. The incremental societal costs were in favour of IMPROVE when comparing CAU (€7382) to IMPROVE (€5808) for a 3-year time horizon. In the optimistic scenario with a 2% 30-day stroke/death risk, the analysis yielded 2.686 QALY for CAU and 2.699 QALY for IMPROVE. The incremental societal costs for a 3-year time horizon were in favour of IMPROVE, with CAU amounting to €5843 compared with €4569 for IMPROVE. Since CEA has a fourfold greater probability of reducing stroke recurrence in the 70–99% stenosis group compared with 50–99% stenosis,^20^ a CAU scenario was explored where only patients with 70–99% stenosis were considered to undergo revascularisation. This scenario was also cost-effective for IMPROVE. It resulted in a QALY of 2.663 for CAU, while the QALY of IMPROVE was 2.692. Societal costs were €4834 for CAU and €5072 for IMPROVE during a 3-year time horizon, resulting in an incremental cost-effectiveness ratio (ICER) of €8302/QALY gain. Deterministic analysis confirmed the findings of these probabilistic scenario analyses.

Subgroups of patients with <50%, 50–69% and 70–99% stenosis were analysed separately to ensure IMPROVE implementation would be beneficial to all stenosis severities (online supplemental table 7). The largest potential of IMPROVE can be seen in patients with <50% of carotid stenosis. Out of the 347 patients with <50% stenosis, on average 18.5 (5.3%) patients would have an ipsilateral ischaemic stroke for CAU and 10.5 (3.0%) patients experienced an ipsilateral ischaemic stroke when treatment selection was dependent on IMPROVE. In the group of 201 patients with 50–69% stenosis, 8.1 versus 5.4 (4.0% vs 2.7%) patients experienced an ipsilateral ischaemic stroke after CAU-based and IMPROVE-based strategies, respectively. Out of the 130 patients with 70–99% stenosis in the pooled dataset, the model estimated that CAU and IMPROVE would lead to an ipsilateral ischaemic stroke in 5.4 for CAU versus 4.6 for IMPROVE (4.2% vs 3.5%) patients during the 3 years after the index event. CEA would be performed in no patients with <50% stenosis under CAU, while IMPROVE would select on average 63.9 (18.4%) out of 347 patients for surgery. All patients with 50–99% stenosis would be selected for the surgical procedure under CAU; however, IMPROVE deemed surgical intervention necessary in only 94.2 (46.9%) patients with 50–69% stenosis and 109.0 (83.8%) patients with 70–99% stenosis. Societal costs of IMPROVE were higher by €1481/patient (95% CI €1466 to €1496) for <50% carotid stenosis, while costs were lower by €5657/patient (95% CI €5619 to €5926) for 50–69% carotid stenosis, and lower by €1788/patient (95% CI €1777 to €1799) for 70–99% carotid stenosis. A minimal QALY gain was identified of 0.017 (95% CI 0.017 to 0.017), 0.019 (95% CI 0.019 to 0.019) and 0.006 (95% CI 0.006 to 0.006) for patients with <50%, 50–69% and 70–99% stenosis, respectively.

### Cost-effectiveness during a life-time horizon

**Figure 4:**
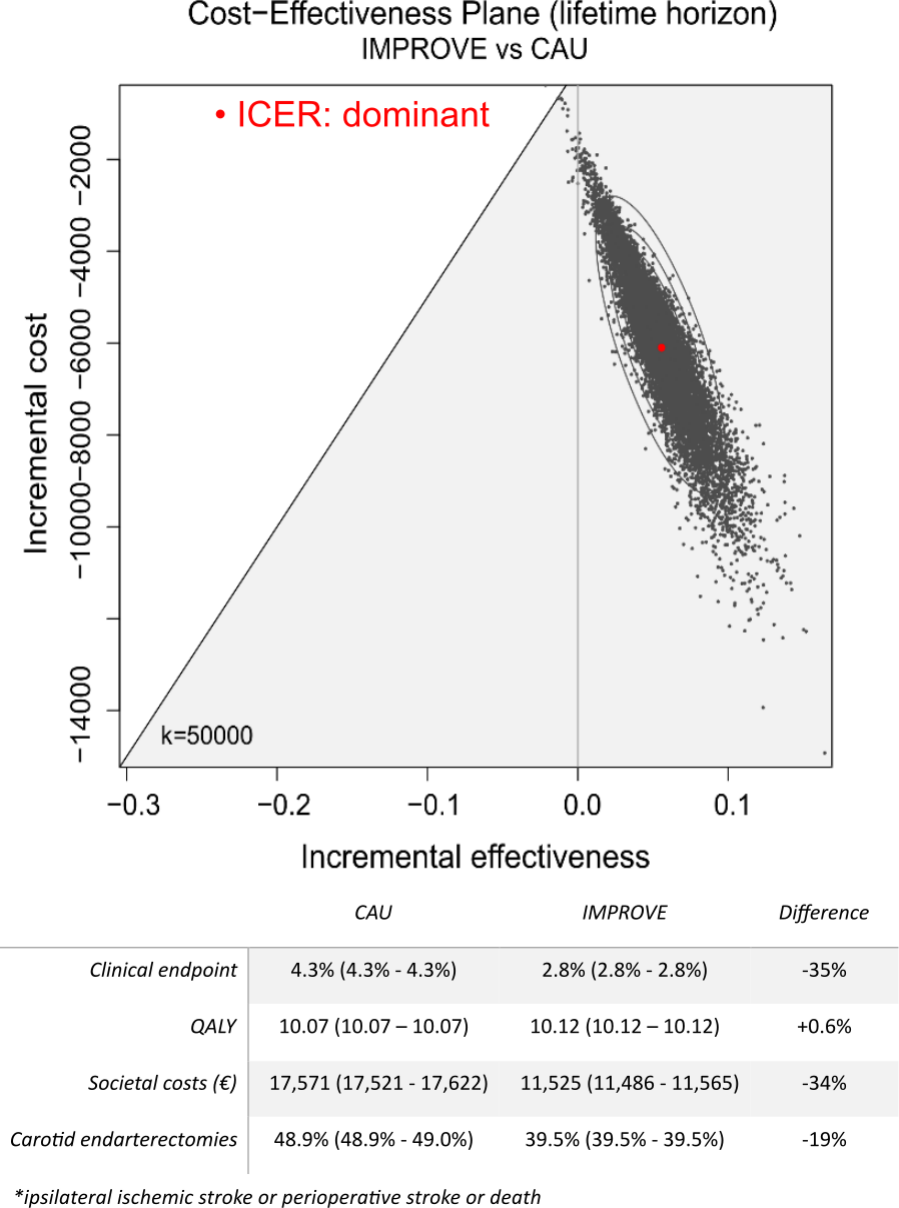
Cost-effectiveness plane of IMPROVE versus CAU for a life-time horizon. Numbers of ipsilateral ischaemic strokes, QALY/patient, societal costs/patient and revascularisations performed are reported with 95% CIs. Index event costs were excluded. CAU, care as usual; ICER, incremental cost-effectiveness ratio; Quality-Adjusted Life Year, QALY.

Applying the mRS-specific lifetime cost and QALYs (online supplemental table 5) to the numbers of patients in each of the mRS scores at the end of the 3-year time horizon resulted in a decrease in life-time societal costs by €6046/patient (95% CI €6015 to €6078) for IMPROVE versus CAU. The average QALY/person remained similar with a marginal increase of 0.056 (95% CI 0.055 to 0.056) QALY over the course of a lifetime for IMPROVE versus CAU. All simulations were cost-effective for IMPROVE with a vast majority in the quadrant of QALY gain and cost-saving (figure 4).

**Figure 5:**
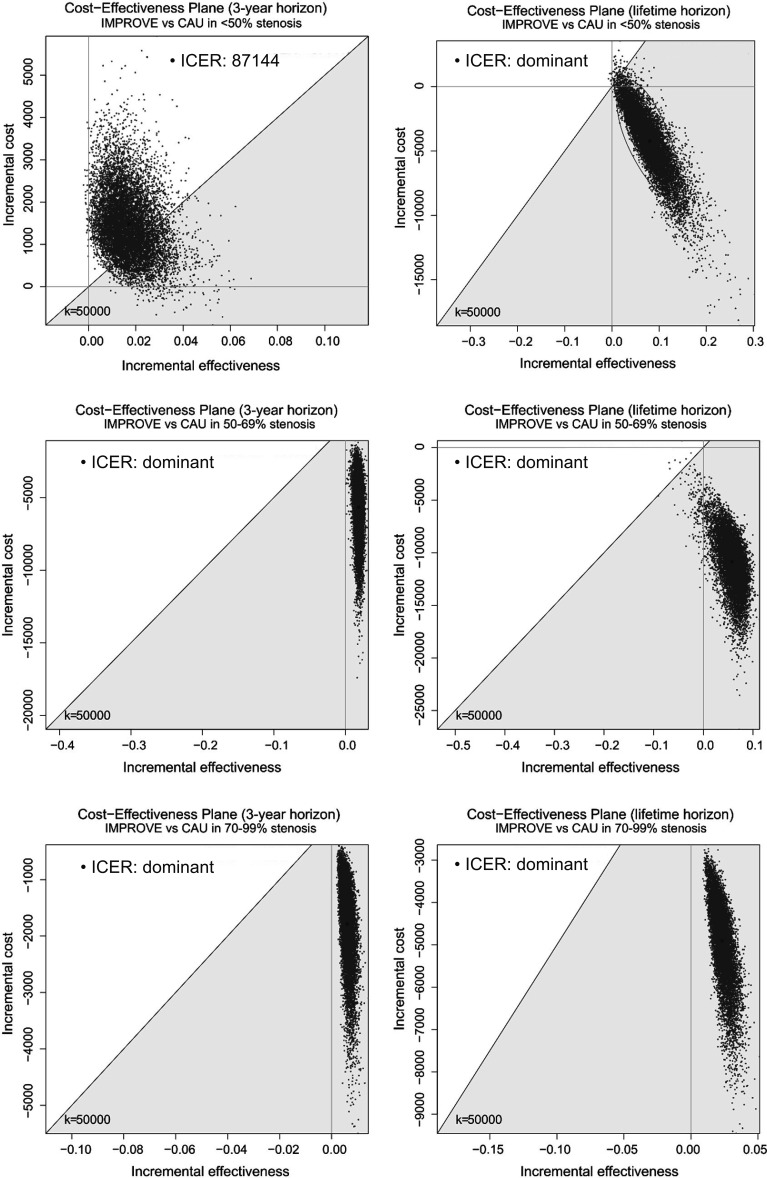
Cost-effectiveness planes of IMPROVE versus CAU for subgroups of degrees of stenosis for 3-year and lifetime horizons. CAU, care as usual; ICER, incremental cost-effectiveness ratio.

Cost-effectiveness of IMPROVE on a lifetime horizon was also estimated per subgroup of the degree of stenosis (online supplemental table 8). While the cost-effectiveness of IMPROVE in patients with <50% stenosis exceeded the willingness-to-pay limit with an ICER of €87 141 for the 3-year time horizon, prolonging the time horizon to lifetime does show a societal cost reduction of €4238/patient (95% CI €4188 to €4289) and an average QALY gain of 0.080 (95% CI 0.079 to 0.081). For patients with 50–69% stenosis, IMPROVE caused a €10 655 (95% CI €10 605 to €10 705) cost reduction and 0.057 (95% CI 0.057 to 0.058) QALY gain compared with CAU. A €4761 (95% CI €4779 to €4 794) cost reduction and 0.023 (0.023–0.023) QALY gain was estimated for patients with >70% stenosis over the course of a lifetime. IMPROVE was cost-effective in all subgroups of carotid stenosis for the life-time horizon (figure 5).

## Discussion

A model-based decision analysis identified IMPROVE-based selection for CEA as a cost-effective strategy to reduce the number of ipsilateral ischaemic strokes and perioperative strokes and deaths in symptomatic patients with carotid artery disease considering a 3-year time horizon. Selection of patients to undergo CEA when their 3-year IMPROVE-estimated ipsilateral ischaemic stroke risk exceeded 7% was superior to a CAU selection strategy of selecting patients with 50–99% stenosis for this intervention. The optimal risk threshold with the maximum ipsilateral ischaemic stroke and perioperative stroke and death reduction of ~35% (CAU: 4.3% vs IMPROVE: 2.8%) was established at a 10% 3-year ipsilateral ischaemic stroke risk according to IMPROVE. Alternative scenario analyses with a higher and lower surgical stroke/death risk of 6% and 2% instead of 3.6% in the base case and an alternative CAU scenario selecting only patients with 70–99% for CEA also resulted in IMPROVE as the preferred strategy. Alternative scenarios also resulted in a similar QALY and reduced societal costs for IMPROVE versus CAU.

While guidelines are clear on the benefit of carotid revascularisation in patients with >70% stenosis, guidelines are less conclusive about the use of the procedure in patients with 50–69% stenosis or below 50% stenosis.^2^ Subgroup analyses also resulted in a beneficial effect of IMPROVE on ipsilateral ischaemic stroke reduction, and consequently, a marginal QALY gain for all subgroups of carotid stenosis, that is, <50%, 50–69% and 70–99%. IMPROVE was already cost-effective within 3 years for patients with 50–99% stenosis with the benefit increasing further on extension to a lifetime horizon. While the number of ipsilateral ischaemic strokes was almost halved for patients with <50% stenosis in the IMPROVE strategy, the ICER did not meet willingness-to-pay criteria on a 3-year time horizon for this subgroup. Considering the increase in revascularisations in patients with <50% stenosis (CAU: 0%, IMPROVE: 18.4%), this result was anticipated. Carotid revascularisations have a procedural risk on short term and reduce the stroke risk on longer term. Extension of the time horizon to lifetime confirmed that IMPROVE is ultimately cost-effective in all subgroups of carotid stenosis, with the vast majority of all analyses in the cost-reductive and QALY-gaining quadrant of the cost-effectiveness plane.

A strength of this decision-analytic study was the use of a pooled dataset with patients who received recent OMT. The dataset strongly represents the target population due to its similarity to large clinical trials.^20^ The performance of both CAU and IMPROVE strategies could be compared in this dataset, since all necessary parameters, including IPH scores on MRI, were available. Multiple imputation was performed on the small number of missing values present in the dataset, reducing bias in comparison to complete-case analysis. Another strength was the determination of cost-effectiveness within subgroups of the degree of stenosis and for several scenarios, which all resulted in IMPROVE as the preferred strategy. The subgroup analyses confirm that the use of IMPROVE for selection for CEA would be justified for patients with mild, moderate and severe stenosis.

Patients with amaurosis fugax are considered symptomatic; however, in practice, some apprehension towards carotid revascularisation exists with some guidelines limiting revascularisation to those with multiple episodes.^28^ The classification of the index event is an important parameter of IMPROVE, meaning that in order for amaurosis fugax patients to be stratified for carotid revascularisation other parameters already placed the patient at high risk for ipsilateral ischaemic stroke. Less than one-third of amaurosis fugax patients were stratified for revascularisation by IMPROVE using the optimised 10% risk threshold, all having IPH-positive plaques and minimal 50% stenosis (3/4 with 70–99% stenosis).

As a limitation of the decision-analytic model, it should be noted that the performance of the IMPROVE model was estimated in a subset of patient data from the derivation cohort of IMPROVE. Therefore, the performance of IMPROVE could be optimistic compared with the performance in an independent dataset. However, probabilistic analyses have incorporated the uncertainty regarding the performance of IMPROVE-based and CAU-based stratification for CEA. These analyses supported that even with a performance at the lower bound of the CI, IMPROVE was superior to CAU-based selection. Nevertheless, the absence of external validation remains a key limitation, and findings should be interpreted with caution pending validation in an independent cohort. Furthermore, probabilistic sensitivity analyses were conducted using 10 000 Monte Carlo iterations, with conclusions based on cost-effectiveness acceptability curves and the probability of cost-effectiveness across willingness-to-pay thresholds, which accounts for joint parameter uncertainty and mitigates the risk of spurious stochastic findings. Nevertheless, external validation of the IMPROVE model is needed to definitively decide on the optimal threshold for stratification for carotid revascularisation before large-scale implementation. Minor variations in the optimal threshold could apply to different cohorts, but the main finding that IMPROVE-based treatment selection has a high probability of being cost-effective likely holds across populations. Another limitation is that healthcare and indirect societal costs could not be separated, as the available cost estimates were retrieved from sources that reported only aggregated values. This limits transparency regarding the relative contribution of each cost component, and future cost-effectiveness studies should separately report these cost types. Last, several clinical prediction tools have previously been developed to estimate stroke risk in patients with symptomatic carotid stenosis, including the Oxford Carotid Stenosis Risk Tool and the adjusted version, the Carotid Artery Risk score. A direct comparison between these tools and the IMPROVE model was not possible in the present study because the Oxford model was developed in patients with ≥50% stenosis and several required predictor variables were not available in our pooled dataset. Future studies using independent external datasets should perform head-to-head comparisons between IMPROVE and established clinical risk tools to further evaluate their relative performance.

In this decision-analytic study, we identified an IMPROVE-guided selection of patients to undergo carotid revascularisation as a cost-effective intervention. These findings confirm the importance of identifying plaque vulnerability with non-invasive MR imaging. Since only half of the patients need an MRI to be categorised in the revascularisation or OMT only group according to IMPROVE, the impact of the MRI scans on healthcare costs and MRI capacity is limited. Furthermore, the required carotid MRI sequence can be incorporated into existing cerebrovascular MRI protocols by extending the scan time by approximately 5 min. The acceptability of the IMPROVE model was previously evaluated among Dutch clinicians involved in carotid revascularisation decision-making. Although some acknowledged the logistical challenge of obtaining MRI before decision-making, the majority expressed support for the model and endorsed its clinical use once the clinical benefit is prospectively validated.^29^

Improvement has shown to improve stratification for CEA by accurately selecting patients most likely to benefit from the intervention. We demonstrated that personalising treatment selection can reduce the cumulative endpoint perioperative stroke and death and ipsilateral stroke recurrence. With this, the IMPROVE model is an add-on to the classic selection criteria ‘symptomatic status’ and ‘degree of stenosis’. Currently, the IMPROVE model is used in research settings only, since its value still needs to be demonstrated in clinical studies. Implementation in routine practice is envisioned as a digital decision-support tool, which needs to be developed in collaboration with relevant stakeholders.

In conclusion, IMPROVE-based selection of patients with symptomatic carotid artery stenosis for CEA can prevent approximately one-third of ipsilateral ischaemic strokes and perioperative strokes and deaths within 3 years compared with CAU. In addition, IMPROVE reduces the number of carotid endarterectomies performed by~20%, while also improving QALY and reducing societal costs. IMPROVE-based patient selection still needs to be validated in a randomised clinical trial.

## Supplementary material

10.1136/bmjopen-2025-114391 online supplemental file 1

## Data Availability

Data are available upon reasonable request.
