# Supplementary material for: Risk-based selection for carotid revascularisation using the IMPROVE score versus standard care in symptomatic carotid artery disease: a model-based cost-effectiveness analysis using pooled-data
Source: BMJ Open. 2026 May 27;16(5):e114391. doi: 10.1136/bmjopen-2025-114391 (PMC13218140; doi:10.1136/bmjopen-2025-114391)
Supplement: online supplemental file 1 [file bmjopen-16-5-s001.docx]

# Supplemental materials

## Supplemental table 1: CHEERS 2022 checklist

|  | **Item** | **Guidance for Reporting** | **Reported in section** |
| --- | --- | --- | --- |
| **TITLE** | | |  |
| Title | 1 | Identify the study as an economic evaluation and specify the interventions being compared. | Page 0 |
| **ABSTRACT** | | |  |
| Abstract | 2 | Provide a structured summary that highlights context, key methods, results and alternative analyses. | Page 1 |
| **INTRODUCTION** | | |  |
| Background and objectives | 3 | Give the context for the study, the study question and its practical relevance for decision making in policy or practice. | Page 2-3 |
| **METHODS** | | |  |
| Health economic  analysis plan | 4 | Indicate whether a health economic analysis plan was developed and where available. | NA |
| Study population | 5 | Describe characteristics of the study population (such as age range, demographics, socioeconomic, or clinical characteristics). | Page 4-5 |
| Setting and location | 6 | Provide relevant contextual information that may influence findings. | Page 4-5 |
| Comparators | 7 | Describe the interventions or strategies being compared and why chosen. | Page 3-4 |
| Perspective | 8 | State the perspective(s) adopted by the study and why chosen. | Page 3 |
| Time horizon | 9 | State the time horizon for the study and why appropriate. | Page 3 |
| Discount rate | 10 | Report the discount rate(s) and reason chosen. | Page 3 |
| Selection of outcomes | 11 | Describe what outcomes were used as the measure(s) of benefit(s) and harm(s). | Page 3 |
| Measurement of outcomes | 12 | Describe how outcomes used to capture benefit(s) and harm(s) were measured. | Page 4-5 |
| Valuation of outcomes | 13 | Describe the population and methods used to measure and value outcomes. | Page 3-5 |
| Measurement and valuation of resources  and costs | 14 | Describe how costs were valued. | Page 3, 6-7 |
| Currency, price date, and conversion | 15 | Report the dates of the estimated resource quantities and unit costs, plus the currency and year of conversion. | Page 7 |
| Rationale and  description of model | 16 | If modelling is used, describe in detail and why used. Report if the model  is publicly available and where it can be accessed. | Page 5-7 |
| Analytics and assumptions | 17 | Describe any methods for analysing or statistically transforming data, any extrapolation methods, and approaches for validating any model used. | Page 7-8 |
| Characterizing heterogeneity | 18 | Describe any methods used for estimating how the results of the study vary for sub-groups. | Page 7-8 |
| Characterizing  distributional effects | 19 | Describe how impacts are distributed across different individuals  or adjustments made to reflect priority populations. | Page 7-8 |
| Characterizing uncertainty | 20 | Describe methods to characterize any sources of uncertainty in the analysis. | Page 7 |
| Approach to engagement with patients and others affected by the study | 21 | Describe any approaches to engage patients or service recipients, the general public, communities, or stakeholders (e.g., clinicians or payers) in the design of the study. | NA |
| **RESULTS** | | |  |
| Study parameters | 22 | Report all analytic inputs (e.g., values, ranges, references) including uncertainty or distributional assumptions. | Page 17-18 |
| Summary of main results | 23 | Report the mean values for the main categories of costs and outcomes of interest and summarise them in the most appropriate overall measure. | Page 9-11 |
| Effect of uncertainty | 24 | Describe how uncertainty about analytic judgments, inputs, or projections  affect findings. Report the effect of choice of discount rate and time horizon, if applicable. | Page 10 |
| Effect of engagement with patients and others affected by the study | 25 | Report on any difference patient/service recipient, general public, community, or stakeholder involvement made to the approach or findings of the study | NA |
| **DISCUSSION** | | |  |
| Study findings, limitations, generalizability, and current knowledge | 26 | Report key findings, limitations, ethical or equity considerations not captured, and how these could impact patients, policy, or practice. | Page 12-13 |
| **OTHER RELEVANT INFORMATION** | | | |
| Source of funding | 27 | Describe how the study was funded and any role of the funder in the identification, design, conduct, and reporting of the analysis | Page 13 |
| Conflicts of interest | 28 | Report authors conflicts of interest according to journal or  International Committee of Medical Journal Editors requirements. | Page 13 |

NA=not applicable

Husereau D, Drummond M, Augustovski F, de Bekker-Grob E, Briggs AH, Carswell C, Caulley L, Chaiyakunapruk N, Greenberg D, Loder E, Mauskopf J, Mullins CD, Petrou S, Pwu RF, Staniszewska S; CHEERS 2022 ISPOR Good Research Practices Task Force. Consolidated Health Economic Evaluation Reporting Standards 2022 (CHEERS 2022) Statement: Updated Reporting Guidance for Health Economic Evaluations. BMJ. 2022;376:e067975.

The checklist is Open Access distributed in accordance with the terms of the Creative Commons Attribution (CC BY 4.0) license, which permits others to distribute, remix, adapt and build upon this work, for commercial use, provided the original work is properly cited. See: [http://creativecommons.org/licenses/by/4.0/.](http://creativecommons.org/licenses/by/4.0/)

## Supplemental figure 1

**The performance of care as usual (CAU) and IMPROVE for predicting ipsilateral ischemic stroke risk**. Patients were categorized at high risk for ipsilateral ischemic stroke when ≥50% degree of stenosis (CAU) or when the calculated 3-year ipsilateral ischemic stroke risk exceeded the respective threshold (IMPROVE). Thresholds of 5-20% 3-year ipsilateral ischemic stroke risk have been assessed.

## Supplemental figure 2


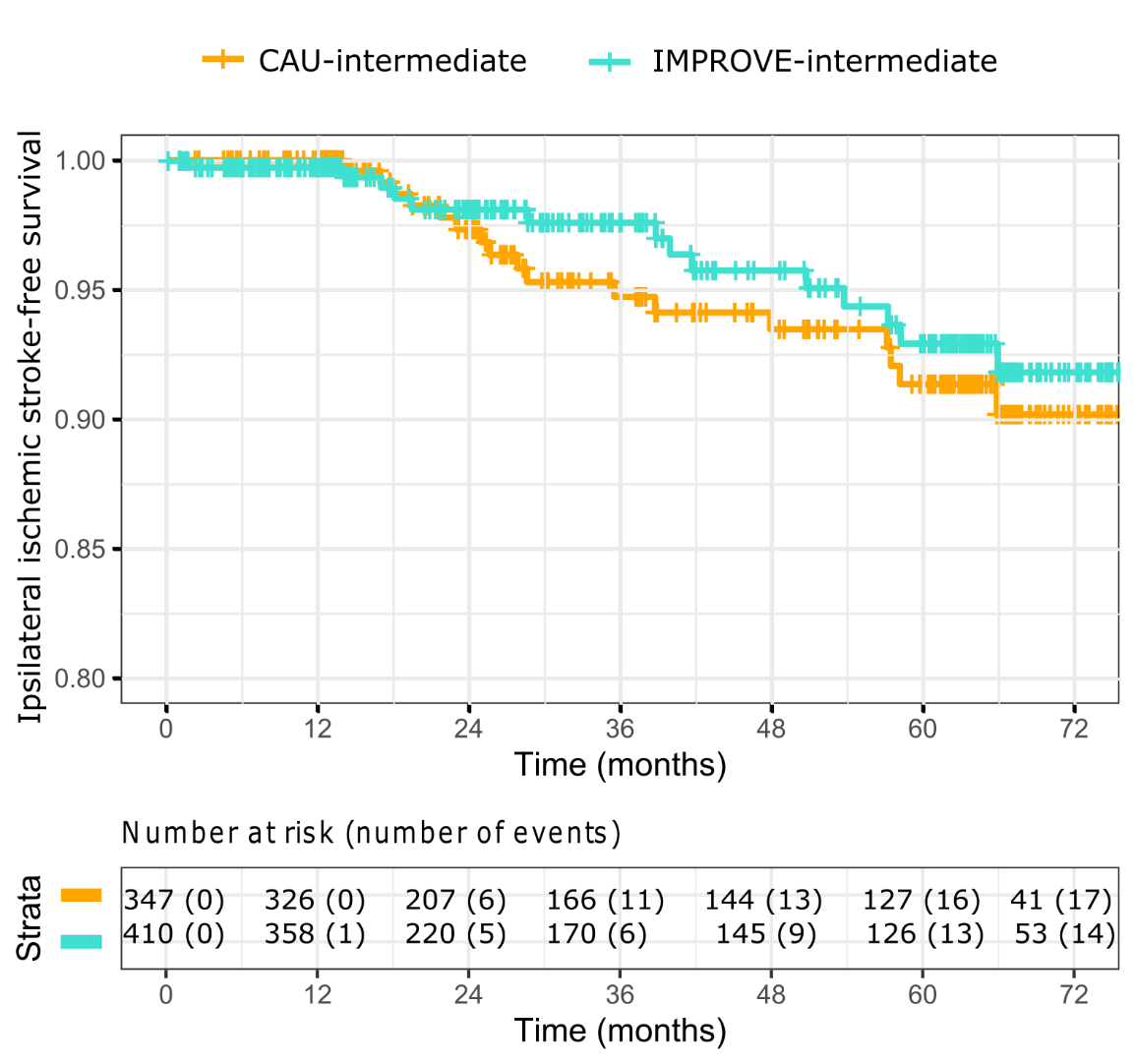


**Kaplan-Meier plot of CAU intermediate risk versus IMPROVE intermediate risk.** Recently symptomatic patients (n=678) were categorized at intermediate risk when having a carotid stenosis <50% stenosis for CAU, and a <10% 3-year ipsilateral ischemic stroke risk for IMPROVE. In the first 3 years the stroke-free survival is higher in the intermediate risk patients that are stratified according to IMPROVE compared to those stratified according to CAU. From 3 to 6 years after the index event, the rate of ipsilateral ischemic stroke recurrence on OMT is similar between CAU and IMPROVE-based stratification.

## Supplemental Table 2

| Base-case VALUE | | | Distribution | source |
| --- | --- | --- | --- | --- |
| CAU | Sensitivity (%) |  | beta (66, 18) | pooled dataset |
|  | Specificity (%) |  | beta (328, 266) | pooled dataset |
| IMPROVE (10%) | Sensitivity (%) |  | beta (77, 9) | pooled dataset |
|  | Specificity (%) |  | beta (401, 190) | pooled dataset |
| Alterative thresholds | | | | |
| CAU (≥ 70%) | Sensitivity (%) |  | beta (35, 50) | pooled dataset |
|  | Specificity (%) |  | beta (499, 95) | pooled dataset |
| IMPROVE (5%) | Sensitivity (%) |  | beta (72, 6) | pooled dataset |
|  | Specificity (%) |  | beta (263, 337) | pooled dataset |
| IMPROVE (6%) | Sensitivity (%) |  | beta (72, 6) | pooled dataset |
|  | Specificity (%) |  | beta (269, 332) | pooled dataset |
| IMPROVE (7%) | Sensitivity (%) |  | beta (72, 6) | pooled dataset |
|  | Specificity (%) |  | beta (280, 320) | pooled dataset |
| IMPROVE (8%) | Sensitivity (%) |  | beta (73, 7) | pooled dataset |
|  | Specificity (%) |  | beta (315, 282) | pooled dataset |
| IMPROVE (9%) | Sensitivity (%) |  | beta (75, 7) | pooled dataset |
|  | Specificity (%) |  | beta (352, 243) | pooled dataset |
| IMPROVE (11%) | Sensitivity (%) |  | beta (76, 17) | pooled dataset |
|  | Specificity (%) |  | beta (438, 147) | pooled dataset |
| IMPROVE (12%) | Sensitivity (%) |  | beta (71, 22) | pooled dataset |
|  | Specificity (%) |  | beta (463, 122) | pooled dataset |
| IMPROVE (13%) | Sensitivity (%) |  | beta (74, 22) | pooled dataset |
|  | Specificity (%) |  | beta (478, 104) | pooled dataset |
| IMPROVE (14%) | Sensitivity (%) |  | beta (72, 22) | pooled dataset |
|  | Specificity (%) |  | beta (485, 98) | pooled dataset |
| IMPROVE (15%) | Sensitivity (%) |  | beta (72, 22) | pooled dataset |
|  | Specificity (%) |  | beta (491, 92) | pooled dataset |
| IMPROVE (16%) | Sensitivity (%) |  | beta (72, 22) | pooled dataset |
|  | Specificity (%) |  | beta (495, 88) | pooled dataset |
| IMPROVE (17%) | Sensitivity (%) |  | beta (71, 23) | pooled dataset |
|  | Specificity (%) |  | beta (497, 87) | pooled dataset |
| IMPROVE (18%) | Sensitivity (%) |  | beta (71, 23) | pooled dataset |
|  | Specificity (%) |  | beta (498, 86) | pooled dataset |
| IMPROVE (19%) | Sensitivity (%) |  | beta (71, 23) | pooled dataset |
|  | Specificity (%) |  | beta (501, 83) | pooled dataset |
| IMPROVE (20%) | Sensitivity (%) |  | beta (71, 23) | pooled dataset |
|  | Specificity (%) |  | beta (505, 79) | pooled dataset |

## Supplemental Table 3

The utilities for the calculation of the quality-adjusted life years were based on the distribution of modified Rankin scores (mRS) at patient discharge ^1^ and the utilities that were reported per mRS ^2^. Below an overview of the distribution of patient mRS scores at discharge and utilities, followed by the calculation of utilities of non-disabling and disabling strokes.

|  | **mRS 0** | **mRS 1** | **mRS 2** | **mRS 3** | **mRS 4** | **mRS 5** |
| --- | --- | --- | --- | --- | --- | --- |
| Distribution | 255 (11%) | 690 (29%) | 268 (11%) | 264 (11%) | 627 (26%) | 285 (12%) |
| Utility | 0.96 (0.02) | 0.90 (0.02) | 0.77 (0.02) | 0.56 (0.04) | 0.35 (0.07) | 0.18 (0.06) |

| **Amaurosis fugax/TIA** | **Non-disabling stroke** | **Disabling stroke** |
| --- | --- | --- |
| mRS 0 = 0.96 | 0.90 * (29 / (29+11)) + 0.77 * (11/ (29+11)) = 0.864 | 0.56 * (11 / (11+26+12)) + 0.35 * (11/ (11+26+12)) +  0.18 * (11/ (11+26+12)) = 0.356 |

For the recurrence of a non-disabling stroke, a disutility of -0.0524 was previously reported ^3^. Therefore, utility of non-disabling stroke recurrence was 0.864 – 0.052 = 0.812.

## Supplemental Table 4

The presence of intraplaque hemorrhage (IPH) on MRI is an important contributor of the IMPROVE model, however an MRI is not always needed to stratify a patient into high or intermediate risk. No MRI was performed in patients for who reclassification based on the outcome of the MRI was not possible since all other predictors would already lead to a high or intermediate risk regardless of IPH presence. Below an overview can be found of the proportion of patients that would need to undergo an MRI to enable risk stratification for each of the evaluated IMPROVE risk thresholds.

| **IMPROVE threshold 3-year ipsilateral ischemic stroke risk** | **MRI needed in ...% of cases** | **Distribution (α,β)** |
| --- | --- | --- |
| 5% | 53.7% | beta (364, 314) |
| 6% | 54.8% | beta (372, 306) |
| 7% | 57.1% | beta (387, 291) |
| 8% | 61.3% | beta (416, 262) |
| 9% | 59.6% | beta (404, 274) |
| 10% | 52.9% | beta (359, 319) |
| 11% | 42.8% | beta (290, 388) |
| 12% | 39.4% | beta (267, 410) |
| 13% | 41.2% | beta (279, 399) |
| 14% | 42.5% | beta (288, 390) |
| 15% | 43.5% | beta (295, 383) |
| 16% | 43.8% | beta (297, 381) |
| 17% | 43.2% | beta (293, 385) |
| 18% | 42.9% | beta (291, 387) |
| 19% | 42.0% | beta (285, 393) |
| 20% | 41.2% | beta (279, 399) |

## Supplemental Table 5

The average mRS-dependent societal costs and QALY per person for a life-time time horizon were calculated based on costs and QALY presented in a recent Dutch cost-effectiveness study of stroke patients ^2^. Costs and QALYs were calculated using an existing stroke model with a starting age of 74 ^4^. Costs were discounted by 4%, while QALYs were discounted by 1.5% in accordance with Dutch guidelines for cost-effectiveness studies.

| **Average life-time costs (€, in 2022)** |
| --- |

| **mRS0** | **mRS1** | **mRS2** | **mRS3** | **mRS4** | **mRS5** |
| --- | --- | --- | --- | --- | --- |
| 55317 | 67647 | 133603 | 474622 | 660937 | 1041095 |

| **Average life-time QALY** | | | | | |
| --- | --- | --- | --- | --- | --- |
| **mRS0** | **mRS1** | **mRS2** | **mRS3** | **mRS4** | **mRS5** |
| 7.771 | 7.435 | 6.556 | 4.816 | 3.115 | 0.795 |

mRS=modified Rankin score

## Supplemental Table 6: Deterministic and probabilistic decision-analytic analyses over a 3-year time horizon.

| **Strategy** | **Ipsilateral ischemic strokes**  **(in n=678 patients)** | | **QALY/person** | |
| --- | --- | --- | --- | --- |
|  | Probabilistic | Deterministic | Probabilistic | Deterministic |
| CAU | 29.0 (21.9-29.1) | 29 | 2.677 (2.676-2.678) | 2.678 |
| 5% | 22.0 (21.9-22.0) | 22 | 2.684 (2.683-2.685) | 2.685 |
| 6% | 21.9 (21.8-21.9) | 22 | 2.684 (2.683-2.685) | 2.685 |
| 7% | 21.5 (21.4-21.5) | 21 | 2.685 (2.676-2.678) | 2.685 |
| 8% | 21.0 (21.0-21.2) | 21 | 2.678 (2.676-2.678) | 2.687 |
| 9% | 19.5 (19.5-19.6) | 19 | 2.689 (2.688-2.690) | 2.689 |
| 10% | 19.1 (19.0-19.2) | 19 | 2.691 (2.690-2.692) | 2.692 |
| 11% | 22.6 (22.6-22.7) | 23 | 2.691 (2.690-2.692) | 2.692 |
| 12% | 25.4 (25.4-25.5) | 25 | 2.691 (2.690-2.692) | 2.691 |
| 13% | 24.4 (24.3-24.5) | 24 | 2.692 (2.692-2.693) | 2.692 |
| 14% | 24.7 (24.6-24.7) | 25 | 2.692 (2.691-2.693) | 2.693 |
| 15% | 24.5 (24.4-24.6) | 24 | 2.692 (2.692-2.693) | 2.268 |
| 16% | 24.4 (24.3-24.5) | 24 | 2.693(2.692-2.693) | 2.693 |
| 17% | 24.5 (24.5-24.6) | 24 | 2.693 (2.692-2.693) | 2.693 |
| 18% | 24.6 (24.5-24.6) | 24 | 2.693 (2.692-2.694) | 2.693 |
| 19% | 24.5 (24.4-24.6) | 24 | 2.693 (2.693-2.694) | 2.693 |
| 20% | 24.4 (24.4-24.5) | 24 | 2.693 (2.692-2.694) | 2.693 |
| **Strategy** | **Societal costs/person (€)** | | **Incremental cost-effectiveness ratio (ICER)** | |
|  | Probabilistic | Deterministic | Probabilistic | Deterministic |
| CAU | 6,509 (6,474-6,543) | 6,493 | reference | reference |
| 5% | 7,310 (7,268-7,352) | 7,283 | 117,336 | 112,072 |
| 6% | 7,219 (7,178-7,260) | 7,194 | 99,928 | 95,434 |
| 7% | 7,040 (7,040-7,081) | 7,017 | 68,298 | 65,419 |
| 8% | 6,501 (6,464-6,538) | 6,480 | Dominant | Dominant |
| 9% | 5,888 (5,855-5,921) | 5,861 | Dominant | Dominant |
| 10% | 5,123 (5,095-5,151) | 5,102 | Dominant | Dominant |
| 11% | 4,487 (4,663-4,711) | 4,669 | Dominant | Dominant |
| 12% | 4,422 (4,401-4,443) | 4,407 | Dominant | Dominant |
| 13% | 4,138 (4,119-4,158) | 4,126 | Dominant | Dominant |
| 14% | 4,054 (4,035-4,073) | 4,041 | Dominant | Dominant |
| 15% | 3,961 (3,943-3,979) | 3,949 | Dominant | Dominant |
| 16% | 3,900 (3,883-3,918) | 3,889 | Dominant | Dominant |
| 17% | 3,879 (3862-3,897) | 3,867 | Dominant | Dominant |
| 18% | 3,862 (3,844-3,879) | 3,850 | Dominant | Dominant |
| 19% | 3,813 (3,796-3,831) | 3,800 | Dominant | Dominant |
| 20% | 3,751 (3,735-3,768) | 3,740 | Dominant | Dominant |

The IMPROVE strategy was considered dominant when QALY and costs were in favor of IMPROVE compared to care as usual. Probabilistic results are presented as mean (95% CI).

## Supplemental Table 7: Probabilistic subgroup decision-analytic analyses over a 3-year time horizon for 10% IMPROVE threshold

| **Strategy** | **Ipsilateral ischemic stroke (n)** | **QALY/person** | **Societal costs/person (€)** | **Revascularizations (n)** | **Incremental cost-effectiveness ratio (ICER)** |
| --- | --- | --- | --- | --- | --- |
| **Subgroup: <50% stenosis (n=347)** | | | | | |
| CAU | 18.5 (18.4-18.6) | 2.721 (2.720-2.721) | 1,346 (1,338-1,354) | 0 | reference |
| IMPROVE | 10.5 (10.4-10.6) | 2.734 (2.734-2.738) | 2,819 (2,804-2,834) | 63.9 (63.8-64.0) | 87,144 |
|  | -40.5% | +0.6% | +109% | +63.9 |  |
| **Subgroup: 50-69% stenosis (n=201)** | | | | | |
| CAU | 8.1 (8.1-8.1) | 2.671 (2.671-2.672) | 11,334 (11,265-11,403) | 201 (201-201) | reference |
| IMPROVE | 5.4 (5.4-5.5) | 2.690 (2.689-2.690) | 5,892 (5,859-5,926) | 94.2 (94.1-94.3) | dominant |
|  | -33.3% | +0.7% | -48% | -53.1% |  |
| **Subgroup: 70-99% stenosis (n=130)** | | | | | |
| CAU | 5.4 (5.4-5.4) | 2.625 (2.624-2.625) | 10,993 (10,927-11,058) | 130 (130-130) | reference |
| IMPROVE | 4.6 (4.6-4.6) | 2.630 (2.630-2.631) | 9,343 (9,288-9,398) | 109 (109-109) | dominant |
|  | 14.8% | +0.2% | -15% | -16% |  |

Results are presented as mean (95% CI).

## Supplemental Table 8: Probabilistic subgroup decision-analytic analyses over a lifetime horizon for 10% IMPROVE threshold

| **Strategy** | **Ipsilateral ischemic strokes*** | **QALY/person** | **Societal costs/person (€)** | **Revascularizations (n)** | **Incremental cost-effectiveness ratio (ICER)** |
| --- | --- | --- | --- | --- | --- |
| **Overall (n=678)*** | | | | | |
| CAU | 29.0 (28.9-29.1) | 10.07 (10.07-10.07) | 17,571 (17,521-17,622) | 331.6 (331.4-331.9) | reference |
| IMPROVE | 19.1 (19.0-19.1) | 10.12 (10.12-10.12) | 11,525 (11,486-11,565) | 267.8 (267.6-268.1) | dominant |
|  | -34% | +0.6% | -34.4% | -19.3% |  |
| **Subgroup: <50% stenosis (n=347)** | | | | | |
| CAU | 18.5 (18.4-18.6) | 10.09 (10.09-10.09) | 13,414 (13,342-13,486) | 0 | reference |
| IMPROVE | 10.5 (10.4-10.6) | 10.17 (10.17-10.17) | 9,176 (9,129-9,223) | 63.9 (63.8-64.1) | dominant |
|  | -40.5% | +0.7% | -31.6% | +63.9 |  |
| **Subgroup: 50-69% stenosis (n=201)** | | | | | |
| CAU | 8.1 (8.1-8.1) | 10.07 (10.07-10.07) | 22,919 (22,839-22,999) | 201 (201-201) | reference |
| IMPROVE | 5.4 (5.4-5.5) | 10.13 (10.13-10.13) | 12,264 (12,214-12,314) | 94.2 (94.1-94.3) | dominant |
|  | -33.3% | +0.5% | -46.5% | -53.1% |  |
| **Subgroup: 70-99% stenosis (n=130)** | | | | | |
| CAU | 5.4 (5.4-5.4) | 10.02 (10.00-10.03) | 22,872 (22,712-23,032) | 130 (130-130) | reference |
| IMPROVE | 4.6 (4.6-4.6) | 10.04 (10.03-10.06) | 18,111 (17,957-18,265) | 109 (109-109) | dominant |
|  | 14.8% | +0.2% | -20.8% | -16% |  |

Only ipsilateral ischemic strokes occurring in the first 3 years of the time horizon have been modeled. The risk of stroke after 3-years was estimated to be similar for both strategies. The cost of the index event was removed from the societal costs/person to give an indication of the costs that are potentially preventable. *Number of total ipsilateral ischemic strokes of overall analysis may vary from the sum of the subgroup analyses due to probabilistic methodology.

## References

1. Crespi V, Braga M, Beretta S, Carolei A, Bignamini A, Sacco S. A practical definition of minor stroke. *Neurol. Sci.* 2013;34:1083-1086

2. Pinckaers FME, Grutters JPC, Huijberts I, Gabrio A, Boogaarts HD, Postma AA, et al. Cost and utility estimates per modified rankin scale score up to 2 years post stroke: Data to inform economic evaluations from a societal perspective. *Value Health*. 2024

3. Buisman LR, Rijnsburger AJ, van der Lugt A, Nederkoorn PJ, Koudstaal PJ, Redekop WK. Cost-effectiveness of novel imaging tests to select patients for carotid endarterectomy. *Health Policy and Technology*. 2019;8:111-117

4. van Leeuwen KG, Meijer FJA, Schalekamp S, Rutten MJCM, van Dijk EJ, van Ginneken B, et al. Cost-effectiveness of artificial intelligence aided vessel occlusion detection in acute stroke: An early health technology assessment. *Insights into Imaging*. 2021;12:133
