# Supplementary material for: Risk-based selection for carotid revascularisation using the IMPROVE score versus standard care in symptomatic carotid artery disease: a model-based cost-effectiveness analysis using pooled-data
Source: BMJ Open. 2026 May 27;16(5):e114391. doi: 10.1136/bmjopen-2025-114391 (PMC13218140; doi:10.1136/bmjopen-2025-114391)
Supplement: Supplementary file 2 [file bmjopen-2025-114391.reviewer_comments.pdf]

PEER REVIEW HISTORY

BMJ Open publishes all reviews undertaken for accepted manuscripts. Reviewers are asked to complete a checklist review form and are provided with free text boxes to elaborate on their assessment. These free text comments are reproduced below.

ARTICLE DETAILS

Title (Provisional)

Risk-Based Selection for Carotid Revascularization Using the IMPROVE Score versus Standard Care in Symptomatic Carotid Artery Disease: A Model-based Cost-Effectiveness Analysis using Pooled-Data

Authors

Nies, Kelly; Ramaekers , Bram; Bierens, Juul; Auer, Dorothee P; Schindler, Andreas; Saam, Tobias; Bos, Daniel; de Jong, P. A.; Nederkoorn, Paul J; de Borst, Gert-Jan; van Oostenbrugge, Robert J.; Joore, Manuela; Kooi, M. Eline; Smits, Luc

---

VERSION 1 - REVIEW

**Reviewer**	**1**
**Name**	**Howard, Dominic**
**Affiliation**	**University of Oxford, Nuffield Department of Clinical Neurology**
**Date**	**21-Dec-2025**
**COI**	**None**

Well written manuscript of a model-based cost-effectiveness analysis (CEA) comparing an IMPROVE-based selection strategy versus Care As Usual (CAU) for patients with symptomatic carotid artery disease.The study is timely and clinically relevant, addressing the uncertainty in guidelines regarding revascularization for moderate stenosis. The authors have adhered to the CHEERS 2022 reporting guidelines, which enhances the transparency.

Main concerns:

1. Many physicians and specialists across the globe use the Oxford carotid stenosis risk tool (https://www.ndcn.ox.ac.uk/divisions/cpsd/carotid-stenosis-tool-1) or adjusted version (CAR score - https://www.sealedenvelope.com/car/) for estimating 1 and 5 year stroke risk for patients with symptomatic carotid stenosis. This risk tool is user friendly and only requires routinely collected clinical data to provide an estimated stroke risk. Has the IMPROVE risk model been shown to be superior to the established Oxford Carotid risk tool in predicting stroke risk?

2. The requirement for carotid plaque MRI to detect the presence of IPH for the IMPROVE risk model, together with the complex formula used to calculate the model, make it unlikely to be used in routine clinical practice. How would this be delivered or used in practice?

**Reviewer**	**2**
**Name**	**Niessen, Louis**
**Affiliation**	**Liverpool School of Tropical Medicine, Health Economics**
**Date**	**13-Feb-2026**
**COI**	**None**

This desk-based economic evaluation compares the effects and cost-effectiveness of carotid endarterectomy base on IMPROVE-based triage against current and some risk-based stratification options with / without OMT. The model used pooled follow-up studies with an average observation period of 17 months with outliers. It is carried out technically well, although somewhat messy with a surplus on detailed information. Most of the methods are standard and ok.I appreciate the inclusion of the operation risk.I am not sure what the BMJ standards are. Also, it is unclear the clinical and economic results are substantial enough to motivate an comparative trial. It might be wiser to do an equivalent trial. After some re-writing the paper would be a valuable publication.

Main points

"Model-based cost-effectiveness" in the title and onwards sounds rather awkward and made me puzzle for a while. The article present prediction results (first paragraph Results), various effect estimates, costs, and cost-effectiveness and cost-utility outcomes in various scenarios. Usually in a more clinical or public health journal a title first present the topic area (the comparison) and next / a subtitle on the methods.

- It is a state-of-the-art uncertainty analysis, yet the results in the figures and in the text are presented without confidence intervals / standard errors, especially in the differences of the differences in the two arms. In Figure 2, as an example, these would indicate if the small QALY gain in a short follow-up is clinically relevant at all. Also, using an unequal effect axis (both side equal length) makes it ugly. Next, I feel uneasy with negative ICERs and even more when they include up to six digits. There is a lot of pseudo-precision. I wonder, with all the uncertainty distributions how one accounts for the risk of a stochastic fallacy.

- Societal costs include household costs. It would be good to present the total societal costs in health care costs and the indirect costs. This is rather common and transparent. This gives an important insight for policy makers and patients.

Minor

- I would recommend pooled-data analysis in the title. This makes this explorativ study unique.

- The depiction of the 50,000 euros (BMJ?) threshold is debatable and is not used nor relevant for the findings as the study claims cost-saving and dominance.

VERSION 1 - AUTHOR RESPONSE

Reviewer #1:

Comments and questions:

1. "Many physicians and specialists across the globe use the Oxford carotid stenosis risk tool (https://www.ndcn.ox.ac.uk/divisions/cpsd/carotid-stenosis-tool-1) or adjusted version (CAR score - https://www.sealedenvelope.com/car/) for estimating 1 and 5 year stroke risk for patients with symptomatic carotid stenosis. This risk tool is user friendly and only requires routinely collected clinical data to provide an estimated stroke risk. Has the IMPROVE risk model been shown to be superior to the established Oxford Carotid risk tool in predicting stroke risk?"

We agree that a comparison of the IMPROVE risk model and Oxford carotid risk tool would be highly relevant for clinicians considering implementation of a new prediction model. However, a valid head-to-head comparison requires application of both models in the same population with (near-)complete availability of all predictor variables. This poses two important challenges: 1) Differences in target population. The Oxford Carotid risk tool was developed in patients with ≥50% carotid stenosis, whereas one of the key innovations of the IMPROVE model is the inclusion of patients across the full stenosis spectrum, including those with <50% stenosis. A direct comparison in our pooled dataset would therefore introduce structural bias, as a substantial subgroup included in IMPROVE falls outside the derivation domain of the Oxford model. 2) Unavailable predictor variables. The pooled dataset used to develop IMPROVE did not contain all predictor variables required for calculation of the Oxford Carotid risk tool/CAR score, such as plaque ulceration and peripheral vascular disease. Therefore, a direct comparison was not feasible within the current study.

Importantly, a robust comparison between prediction models should ideally be performed in an independent external dataset that was not used for the development of either model, to avoid optimism bias. We agree that such a comparison would be valuable and have now explicitly stated in the revised manuscript that future external validation studies should include head-to-head comparisons with established clinical tools such as the Oxford Carotid risk tool.

While a direct comparison was not possible, prior validation studies of the Oxford tool have reported only moderate discrimination. In contrast, the IMPROVE model demonstrated good discrimination and calibration in internal validation and uniquely incorporates plaque vulnerability (intraplaque hemorrhage, IPH on MRI), which has repeatedly shown strong independent associations with stroke risk. We have clarified this in the discussion while avoiding overstatement of superiority:

“Last, several clinical prediction tools have previously been developed to estimate stroke risk in patients with symptomatic carotid stenosis, including the Oxford Carotid Stenosis Risk Tool and the adjusted version, the CAR score. A direct comparison between these tools and the IMPROVE model was not possible in the present study because the Oxford model was developed in patients with ≥50% stenosis and several required predictor variables were not available in our pooled dataset. Future studies using independent external datasets should perform head-to-head comparisons between IMPROVE and established clinical risk tools to further evaluate their relative performance.”

2. “The requirement for carotid plaque MRI to detect the presence of IPH for the IMPROVE risk model, together with the complex formula used to calculate the model, make it unlikely to be used in routine clinical practice. How would this be delivered or used in practice?”

Regarding the use of carotid plaque MRI, we acknowledge that this modality is not yet universally embedded in routine clinical care. However, MRI-based plaque characterization, including detection of intraplaque hemorrhage (IPH), is increasingly available in tertiary stroke centers and can be performed using relatively short, non-contrast sequences. Adding a dedicated MRI sequence to detect IPH extends a standard neuro MRI examination with approximately 5 minutes. Alternatively, a stand-alone MRI examination would take about 20 minutes. Importantly, our cost-effectiveness results demonstrate that despite the costs of an additional MRI examination, the IMPROVE-based strategy leads to cost-savings at both 3-year and lifetime horizons. The impact on MRI capacity is further mitigated by the fact that only approximately half of patients require MRI to be categorized into either the revascularization or optimal medical therapy group under IMPROVE. For the other patients, an IPH-positive or IPH-negative MRI scan does not affect the risk category. Recently, we have investigated the acceptance of patient selection for revascularisation using the IMPROVE risk model among stroke clinicians in the Netherlands. While some acknowledged the challenge of incorporating an additional MRI prior to carotid revascularization decision-making, the majority expressed support for the model and endorsed its use once validated in prospective clinical studies. We have now clarified these points in the discussion:

“Furthermore, the required carotid MRI sequence can be incorporated into existing cerebrovascular MRI protocols by extending the scan time with approximately five minutes. The acceptability of the IMPROVE model was previously evaluated among Dutch clinicians involved in carotid revascularization decision-making. Although some acknowledged the logistical challenge of obtaining MRI before decision-making, the majority expressed support for the model and endorsed its clinical use once the clinical benefit is prospectively validated (28).”

With respect to the model’s complexity, we agree that manual calculation would be impractical. However, contemporary clinical prediction models are rarely used as paper-based formulas. We envision IMPROVE being delivered as a digital decision-support tool in which routinely collected clinical variables and imaging findings are entered (or automatically extracted), after which the predicted 3-year stroke risk is generated instantly. Such integration would eliminate computational burden for clinicians. At present, the IMPROVE risk model is only used in research settings to validate the findings presented in this cost-

effectiveness study. The manner of implementation of the tool is a work-in-progress involving relevant stakeholders. We have added this to the discussion:

“Currently, the IMPROVE model is used in research settings; implementation in routine practice is envisioned as a digital decision-support tool, the development of which is ongoing in collaboration with relevant stakeholders.”

Reviewer #2:

Comments and questions:

1. "Model-based cost-effectiveness" in the title and onwards sounds rather awkward and made me puzzle for a while. The article present prediction results (first paragraph Results), various effect estimates, costs, and cost-effectiveness and cost-utility outcomes in various scenarios. Usually in a more clinical or public health journal a title first present the topic area (the comparison) and next / a subtitle on the methods.

Thank you for your suggestion. We have changed our title to the following: “Risk-Based Selection for Carotid Revascularization Using the IMPROVE Score versus Standard Care in Symptomatic Carotid Artery Disease: A Cost-Effectiveness Analysis based on Pooled-Data”.

2. It is a state-of-the-art uncertainty analysis, yet the results in the figures and in the text are presented without confidence intervals / standard errors, especially in the differences of the differences in the two arms. In Figure 2, as an example, these would indicate if the small QALY gain in a short follow-up is clinically relevant at all. Also, using an unequal effect axis (both side equal length) makes it ugly. Next, I feel uneasy with negative ICERs and even more when they include up to six digits. There is a lot of pseudo-precision. I wonder, with all the uncertainty distributions how one accounts for the risk of a stochastic fallacy.

Thank you for your feedback on the uncertainty analyses. We have added the confidence intervals to the uncertainty analyses in both the manuscript and supplementary tables. The addition of 95% confidence intervals required recalculating differences in outcomes at the simulation level rather than from strategy means, which is the statistically correct approach for probabilistic analyses and resulted in minor changes to some of the results.
 We have carefully considered your suggestion regarding the figure axes and presentation. After evaluating alternative layouts, we concluded that the current axis scaling provides the clearest representation of the simulation results and allows the relevant differences between strategies to remain visible. We therefore retained the current format while improving the presentation of uncertainty and removing unnecessary numerical precision in the manuscript. We also agree with your comment about the ICERs. We agree that the negative ICER values in Figure 2, 3 and 5 can be confusing because a negative ICER can refer to a Dominant intervention (cheaper and more effective) or a Dominated intervention (more expensive and less effective). We have therefore replaced negative ICERs in Figure 2, 3, and 5 by the term “dominant”.

Similarly, we agree that reporting excessive digits implies pseudo-precision. Regarding the related concern about stochastic fallacy, our probabilistic sensitivity analysis was conducted

using 10,000 Monte Carlo iterations confirmed to yield stable, converged estimates, and conclusions are based on cost-effectiveness acceptability curves and the probability of cost-effectiveness across willingness-to-pay thresholds rather than point estimates, which inherently accounts for uncertainty and mitigates the risk of stochastic findings. We have added this in the manuscript:

"These analyses supported that even with a performance at the lower bound of the confidence interval, IMPROVE was superior to CAU-based selection. Furthermore, probabilistic sensitivity analyses were conducted using 10,000 Monte Carlo iterations, with conclusions based on cost-effectiveness acceptability curves and the probability of cost-effectiveness across willingness-to-pay thresholds, which accounts for joint parameter uncertainty and mitigates the risk of spurious stochastic findings."

3. Societal costs include household costs. It would be good to present the total societal costs in health care costs and the indirect costs. This is rather common and transparent. This gives an important insight for policy makers and patients.
Thank you for your suggestion. We agree that it is preferred to present the healthcare costs and indirect costs. Unfortunately, the input parameters regarding the cost of a fatal stroke were retrieved from reliable and recent sources that, unfortunately, did not separately report these different types of costs. Therefore, we are not able to add this split of cost types in our manuscript. A future cost-effectiveness study should take into account this split of cost types. We have added the following sentence to our discussion:

"A final limitation of the present study is that healthcare and indirect societal costs could not be separated because the available cost estimates reported aggregated values. A future cost-effectiveness study should take into account this split of cost types."

Minor
1. I would recommend pooled-data analysis in the title. This makes this explorativ study unique.

Thank you for your valuable suggestion. We have changed our title to the following:
"Risk-Based Selection for Carotid Revascularization Using the IMPROVE Score versus Standard Care in Symptomatic Carotid Artery Disease: A Pooled-Data Cost-Effectiveness Analysis"

2. The depiction of the 50,000 euros (BMJ?) threshold is debatable and is not used nor relevant for the findings as the study claims cost-saving and dominance.

We agree that the 50,000 euros threshold does not have a large impact on the majority of demonstrated results. The willingness-to-pay threshold is used as this is the severity-adjusted cost-effectiveness threshold set by the Dutch National Health Care Institute. However, although sparingly, there are several simulations that were not in the dominant quadrant of the cost-effectiveness plane. Especially for the subgroup analysis, the willingness-to-pay threshold was relevant since IMPROVE-based risk stratification for carotid revascularization was not cost-effective in patients with <50% stenosis looking at a 3-year

time horizon. Therefore, we deem the illustration of a willingness-to-pay threshold necessary.

VERSION 2 - REVIEW

Reviewer **1**

Name **Howard, Dominic**

Affiliation **University of Oxford, Nuffield Department of Clinical Neurology**

Date **03-Apr-2026**

COI

The paper has been improved and the revised manuscript is considerably stronger than the original. My main remaining concerns are:

1. The stroke risk estimation for the study is based on 10 year old Dutch registry and audit data. Contemporary studies report much lower procedural stroke and death rates (2% rather than 3.6%), as these risks have reduced considerably over the last 2 decades. The actual benefits of the improve-based triage may therefore be overestimated.

2. It is still not completely clear to the reader, when reading the title and the abstract, that this is modelling analysis without any real-life outcomes. A reader could easily think that actual outcomes of the cohort of 678 patients were being reported. In the title, I would add 'modelling' in front of analysis. In the abstract I would add to the conclusion; 'In this modelling analysis, …'

3. Overall, the study is well performed and well written, but the improve model is fitted to the dataset with no external validation so it is clearly going to perform well. This is only acknowledged briefly in the discussion, and probably should be noted in the methods also. Also, how was a cohort of 678 patients selected? Was there a paper calculation for the cohort size required to determine a difference between CAU and improve?

Reviewer **2**

Name **Niessen, Louis**

Affiliation **Liverpool School of Tropical Medicine, Health Economics**

Date **26-Mar-2026**

COI

The authors responded well to the review comments. It is a pity household costs and health services costs cannot be separated. It is an important lack of transparency.

VERSION 2 - AUTHOR RESPONSE

Reviewer #1:
The paper has been improved and the revised manuscript is considerably stronger than the original. My main remaining concerns are:
1. The stroke risk estimation for the study is based on 10 year old Dutch registry and audit data. Contemporary studies report much lower procedural stroke and death rates (2% rather than 3.6%), as these risks have reduced considerably over the last 2 decades. The actual benefits of the improve-based triage may therefore be overestimated.

We thank the reviewer for this suggestion. To address this concern, we conducted an additional sensitivity analysis using an optimistic scenario in which the 30-day surgical stroke/death risk was set at 2%, reflecting contemporary low perioperative risk estimates. The results did not change substantially: IMPROVE remained the superior strategy across both QALY outcomes and societal costs. The average cost reduction was slightly lower compared to the base-case scenario (-€1,274 versus -€1,386).

The following text has been added to the Methods and Results sections accordingly:

"Two alternative scenarios were modelled: one in which this risk was set at 6%, representing the maximum accepted threshold for procedural risk, and one in which it was set at 2%, reflecting an optimistic scenario characterized by low perioperative risks."

"In the optimistic scenario with a 2% 30-day stroke/death risk, the analysis yielded 2.686 QALY for CAU and 2.699 QALY for IMPROVE. The incremental societal costs for a 3-year time horizon were in favor of IMPROVE, with CAU amounting to €5,843 compared to €4,569 for IMPROVE."

2. It is still not completely clear to the reader, when reading the title and the abstract, that this is modelling analysis without any real-life outcomes. A reader could easily think that actual outcomes of the cohort of 678 patients were being reported. In the title, I would add 'modelling' in front of analysis. In the abstract I would add to the conclusion; 'In this modelling analysis, …'

We thank the reviewer for this suggestion. As recommended, we have added "model-based" and "modelling" in the title and the abstract, respectively.

3. Overall, the study is well performed and well written, but the improve model is fitted to the dataset with no external validation so it is clearly going to perform well. This is only acknowledged briefly in the discussion, and probably should be noted in the methods also. Also, how was a cohort of 678 patients selected? Was there a paper calculation for the cohort size required to determine a difference between CAU and improve?

We thank the reviewer for this important observation. We agree that the lack of external validation is a meaningful limitation that deserves greater prominence. We have added a sentence to the Methods acknowledging that the IMPROVE model was evaluated in a subset of its derivation cohort, and that performance may therefore be optimistic compared to an independent dataset. This point was already noted in the Discussion but has been strengthened accordingly:

"As a limitation of the decision-analytic model, it should be noted that the performance of the IMPROVE model was estimated in a subset of patient data from the derivation cohort of IMPROVE. Therefore, the performance of IMPROVE could be optimistic compared to the performance in an independent dataset. However, probabilistic analyses have incorporated the uncertainty regarding the performance of IMPROVE-based and CAU-based stratification for carotid endarterectomy. These analyses supported that even with a performance at the lower bound of the confidence interval, IMPROVE was superior to CAU-based selection. Nevertheless, the absence of external validation remains a key limitation, and findings should be interpreted with caution pending validation in an independent cohort."

Regarding the cohort size: the 678 patients represent all available data from four cohorts with both carotid MRI and follow-up outcome data on OMT only. No formal sample size calculation was performed, as this was not a prospective study powered to detect a difference between strategies, but rather a decision-analytic modelling study using all eligible existing data. We have clarified this in the Methods:

"The cohort of 678 patients represented all available data from cohorts with both carotid MRI and clinical outcome data under OMT only. No formal sample size calculation was performed, as this was a model-based study utilizing all eligible existing data rather than a prospective study powered to detect a difference between strategies."

Reviewer #2:

The authors responded well to the review comments. It is a pity household costs and health services costs cannot be separated. It is an important lack of transparency.

We thank the reviewer for the positive assessment. We agree that the inability to separate healthcare from indirect societal costs reduces transparency, and have revised the limitation accordingly:

"Another limitation is that healthcare and indirect societal costs could not be separated, as the available cost estimates were retrieved from sources that reported only aggregated values. This limits transparency regarding the relative contribution of each cost component, and future cost-effectiveness studies should separately report these cost types."
